# Supplementary material for: High-resolution spatiotemporal measurement of air and environmental noise pollution in Sub-Saharan African cities: Pathways to Equitable Health Cities Study protocol for Accra, Ghana
Source: BMJ Open. 2020 Aug 20;10(8):e035798. doi: 10.1136/bmjopen-2019-035798 (PMC7440835; doi:10.1136/bmjopen-2019-035798)
Supplement: Supplementary data [file bmjopen-2019-035798supp001.pdf]

High-resolution spatiotemporal measurement of air and environmental noise pollution in sub-Saharan African cities: Pathways to Equitable Health Cities Study protocol for Accra, Ghana

Supplementary Information 1

Quality Assurance and Quality Control Protocol

The field team calibrate equipment prior to each use. Specifically, the UPAS mass flow sensor maintains a steady sampling flow rate over time by internally measuring changes in pressure drop across the filter media. But as part of our quality assurance process, the flow rates are manually checked with a TSI Mass Flowmeter (4000 Series) for possible flow drift prior to and immediately after each monitoring session. Monitors are adjusted as necessary prior to the next deployment. Following a previous protocol used in the same setting [1], samples will be considered valid only if the average flow rate is within 10% of the intended rate of 1 lpm, and the UPAS operated for ≥ 85% of the 7-day measurement period. Additionally, the SLMs are calibrated prior to each monitoring session with a CA114 sound calibrator at 94.0 dB ±0.3 dB and 1000Hz ±0.5% (Convergence Instruments, Canada). If an instrument is consistently reading a calibration offset ±1 dBA, the SLM is pulled out of commission and tested and the data from that session considered invalid.

In order to understand the extent of potential filter and diffusion pad contamination from handling procedures, we collect field blanks at 20% of our sites for filter based $PM_{2.5}$ and NO_x and NO_2 samples. Blank $PM_{2.5}$ samples are prepared as regular samples in the field lab, brought to the field sites, and deployed in the same way as the regular sample, but without the pump being turned on. NO_x/NO_2 blanks are brought to the field sites but not exposed to air in their sealed canisters. During analysis, information from the blank samples will be used to account for residual contamination from the laboratory work, transportation, and field handling processes, which in a previous study in Accra was minimal [1]. We will assess the mean absolute difference of the pre- and post-sampling weights of the blank samples; mean weights within 10 ug will be considered valid [1]. Also, final filters weights will be checked against the limit of detection, computed using the blanks, to be sure all valid samples are above this limit.

We will assess the accuracy and precision of our monitors by conducting **pre-campaign** side-by-side monitoring sessions between all our instruments of the same type (precision) and our instruments next to reference grade or higher-grade monitors (accuracy).

- Prior to field deployment, we tested minute-by-minute monitor-monitor precision for the continuous $PM_{2.5}$ monitors by running all of our monitors alongside each other over a 24-hour period at the University of Ghana, Legon campus, with average relative humidity (RH) (~ 78%) and temperature (29 °C) representative of the city. The continuous $PM_{2.5}$ measurements had good agreement and were within 2-3 ug/m^3 of each other. The continuous $PM_{2.5}$ ZeFan monitor uses the Plantower sensor (model PMS7003) which has been validated in previous studies against a TEOM 1400a analyser and tested for durations ranging from 6 months to a year in various environmental conditions [2,3].
- The filter-based UPAS monitor has been evaluated in previous laboratory and field settings against a federal reference monitor (URG-2000-30EGN-A; URG Corp., USA), personal environmental monitor (PEM 761 - 203; SKC, Inc., USA) and Harvard Impactors, respectively

and has proven valid for ambient, household, and personal monitoring in a typical tropical climate as our study [4–6].

- Our pre-campaign tests of SLM monitor-monitor precision showed good agreement. There was only a 0.5 dBA difference between the monitoring period median values ($LAeq_{1min}$) for 50% of monitors within the IQR bounds around the overall median (25%-75%) and a 1.7 dBA difference between the two monitors with the highest and lowest monitoring period median values. The monitor-monitor precision test was done in Accra and SLMs were exposed 16hrs to multiple sound environments similar to what we would expect during the full monitoring campaign. Our Type II Noise Sentry SLMs were also validated in a separate aircraft noise study conducted in San Francisco against a Type I industry standard instrument (DUO 01dB) [7], and the agreement was high (mean and median second by second difference between the instruments was -0.42 and -0.38 dBA, respectively).

In addition to the pre-campaign monitor-monitor precision tests and accuracy checks, we will collect duplicate samples at 20% of our sites and conduct **mid and post-campaign** precision tests to check their sensitivity over time and accuracy checks with reference grade monitors.

- To understand the extent to which each type of monitor provides consistent measurements among all the units used in the campaign, we are also collecting duplicate samples from co-located instruments at 20% of our rotating measurement sites. Duplicate samples will be evaluated from 20% of sites during the course of the campaign and faulty and malfunctioning instruments will be pulled from the field and data potentially removed from analysis if mean absolute difference between duplicate measurement is > 10 ug/m^3 [1] or >2 dBA ($LAeq_{24hr}$).
- We will additionally co-locate all of our monitors side-by-side for mid and post campaign precision tests for a 1-week period to assess instrument drift over time. Data will be considered invalid if the mean absolute difference between daily/ weekly $PM_{2.5}$ and $LAeq_{24hr}$ measurements differ by > 10 ug/m^3 [1] or >2 dBA.
- Since light-scattering techniques only infer PM mass from detecting particle number concentrations and are impacted by weather conditions (i.e. RH and temperature), their estimates of mass concentration are inexact. Thus, we will co-locate the ZeFan monitors with a U.S. federal equivalent continuous monitor Met One BAM 1020 at three sites, each with unique source influence in Accra for a week at the end of the campaign and adjust the minute-by-minute continuous PM records for impact of relative humidity and then their average against the co-located integrated $PM_{2.5}$ concentrations from UPAS.

The real-time data will be inspected weekly by the field team as it is downloaded from the instruments. Potential implausible values will be identified by inspecting all values that are 5-standard deviations above or below the site and day (or week for filter-based $PM_{2.5}$ and NO_x/NO_2) specific mean value. For the filter based $PM_{2.5}$ data, potentially implausible values will be checked against the monitor run time, weighed mass value, and flow rate. The log sheets will be checked to see if any information on instrument malfunction or other irregularities was noted for the continuous $PM_{2.5}$ and SLM monitors. Values deemed erroneous will be dropped from analysis. Additionally, since monitors are swapped every week, sometimes an entire week of data might be erroneous if the instrument is malfunctioning or if calibration did not occur correctly. We will identify outlier weeks by plotting timeseries of a month worth of data to identify any potential implausible weeks of data and conduct instrument checks, review log sheets, and drop or correct data as needed. Finally, all real-time instruments will have their first 5 minutes of data dropped to allow the instruments to stabilize and the data further trimmed to match the exact monitoring session start and end date and time as recorded by the field team on the data log forms.

References

1 Dionisio K, Arku RE, Hughes AF, *et al.* Air Pollution in Accra Neighborhoods: Spatial, Socioeconomic, and Temporal Patterns. *Environ Sci Technol* 2010;**44**:2270–6. doi:10.1021/es903276s

2 Bulot FMJ, Johnston SJ, Basford PJ, *et al.* Long-term field comparison of the performances of multiple low-cost particulate matter sensors in an urban area. *Sci Rep* 2019;:1–16. doi:10.5281/ZENODO.2531601

3 Malings C, Tanzer R, Hauryliuk A, *et al.* Fine particle mass monitoring with low-cost sensors: Corrections and long-term performance evaluation. *Aerosol Sci Technol* 2019;**0**:1–15. doi:10.1080/02786826.2019.1623863

4 Volckens J, Quinn C, Leith D, *et al.* Development and evaluation of an ultrasonic personal aerosol sampler. *Indoor Air* 2017;**27**:409–16. doi:10.1111/ina.12318

5 Arku RE, Birch A, Shupler M, *et al.* Characterizing exposure to household air pollution within the Prospective Urban Rural Epidemiology (PURE) study. *Environ Int* 2018;**114**:307–17. doi:10.1016/j.envint.2018.02.033

6 Pillarisetti A, Carter E, Rajkumar S, *et al.* Measuring personal exposure to fine particulate matter (PM 2.5) among rural Honduran women: A field evaluation of the Ultrasonic Personal Aerosol Sampler (UPAS). *Environ Int* 2019;**123**:50–3. doi:10.1016/j.envint.2018.11.014

7 Rindleisch TC. A comparative evaluation of two sound level meters. 2018. doi:10.13140/RG.2.2.26395.31520
