# Supplementary material for: High-resolution spatiotemporal measurement of air and environmental noise pollution in Sub-Saharan African cities: Pathways to Equitable Health Cities Study protocol for Accra, Ghana
Source: BMJ Open. 2020 Aug 20;10(8):e035798. doi: 10.1136/bmjopen-2019-035798 (PMC7440835; doi:10.1136/bmjopen-2019-035798)
Supplement: Author's manuscript [file bmjopen-2019-035798.draft_revisions.pdf]

BMJ Open

BMJ Open is committed to open peer review. As part of this commitment we make the peer review history of every article we publish publicly available.

When an article is published we post the peer reviewers' comments and the authors' responses online. We also post the versions of the paper that were used during peer review. These are the versions that the peer review comments apply to.

The versions of the paper that follow are the versions that were submitted during the peer review process. They are not the versions of record or the final published versions. They should not be cited or distributed as the published version of this manuscript.

BMJ Open is an open access journal and the full, final, typeset and author-corrected version of record of the manuscript is available on our site with no access controls, subscription charges or pay-per-view fees (http://bmjopen.bmj.com).

If you have any questions on BMJ Open's open peer review process please email info.bmjopen@bmj.com

BMJ Open

High-resolution assessment of air and environmental noise pollution in sub-Saharan African cities: Pathways to Equitable Health Cities Study protocol for Accra, Ghana

Journal:	*BMJ Open*
Manuscript ID	bmjopen-2019-035798
Article Type:	Protocol
Date Submitted by the Author:	15-Nov-2019
Complete List of Authors:	Clark, Sierra; Imperial College London, School of Public Health<br>Alli, Abosede; University of Massachusetts Amherst, Department of Environmental Health Sciences<br>Brauer, Michael; The University of British Columbia, School of Population and Public Health<br>Ezzati, Majid; Imperial College London, School of Public Health; Imperial College London, MRC-PHE Center for Environment and Health<br>Baumgartner, Jill ; McGill University, Institute for Health and Social Policy; McGill University, Department of Epidemiology, Biostatistics, and Occupational Health<br>Toledano, Mireille; Imperial College London, School of Public Health; Imperial College London, MRC-PHE Center for Environment and Health<br>Hughes, Allison; University of Ghana, Department of Physics<br>Moses, Josephine; University of Ghana, Department of Physics<br>Terkpertey, Solomon; University of Ghana, Department of Physics<br>Nimo, James; University of Ghana, Department of Physics<br>Vallarino, Jose; Harvard University, T.H. Chan School of Public Health<br>Agyei-Mensah, Samuel; University of Ghana, Department of Geography and Resource Development<br>Agyemang, Ernest; University of Ghana, Department of Geography and Resource Development<br>Nathvani, Ricky; Imperial College London, School of Public Health<br>Muller, Emily; Imperial College London, School of Public Health<br>Wang, Jiayuan; University of Massachusetts Amherst, Department of Environmental Health Sciences<br>Beddows, Andrew; King's College London, Department of Analytical, Environmental and Forensic Sciences<br>Kelly, Frank; King's College London, Department of Analytical, Environmental and Forensic Sciences<br>Barratt, Benjamin; King's College London, Department of Analytical, Environmental and Forensic Sciences<br>Arku, Raphael; University of Massachusetts Amherst, Department of Environmental Health Sciences
Keywords:	STATISTICS & RESEARCH METHODS, EPIDEMIOLOGY, PUBLIC HEALTH

For peer review only - http://bmjopen.bmj.com/site/about/guidelines.xhtml

I, the Submitting Author has the right to grant and does grant on behalf of all authors of the Work (as defined in the below author licence), an exclusive licence and/or a non-exclusive licence for contributions from authors who are: i) UK Crown employees; ii) where BMJ has agreed a CC-BY licence shall apply, and/or iii) in accordance with the terms applicable for US Federal Government officers or employees acting as part of their official duties; on a worldwide, perpetual, irrevocable, royalty-free basis to BMJ Publishing Group Ltd ("BMJ") its licensees and where the relevant Journal is co-owned by BMJ to the co-owners of the Journal, to publish the Work in this journal and any other BMJ products and to exploit all rights, as set out in our licence.

The Submitting Author accepts and understands that any supply made under these terms is made by BMJ to the Submitting Author unless you are acting as an employee on behalf of your employer or a postgraduate student of an affiliated institution which is paying any applicable article publishing charge ("APC") for Open Access articles. Where the Submitting Author wishes to make the Work available on an Open Access basis (and intends to pay the relevant APC), the terms of reuse of such Open Access shall be governed by a Creative Commons licence – details of these licences and which Creative Commons licence will apply to this Work are set out in our licence referred to above.

Other than as permitted in any relevant BMJ Author's Self Archiving Policies, I confirm this Work has not been accepted for publication elsewhere, is not being considered for publication elsewhere and does not duplicate material already published. I confirm all authors consent to publication of this Work and authorise the granting of this licence.

For peer review only

High-resolution assessment of air and environmental noise pollution in sub-Saharan African cities: Pathways to Equitable Health Cities Study protocol for Accra, Ghana

Sierra N Clark [a], Abosede S Alli [b], Michael Brauer [c], Majid Ezzati [a,d], Jill Baumgartner [e,f], Mireille Toledano [a,d], Allison Hughes [g], Josephine Moses [g], Solomon Terkpertey [g], James Nimo [g], Jose Vallarino [h], Samuel Agyei-Mensah [i], Ernest Agyemang [i], Ricky Nathvani [a], Emily Muller [a] , Jiayuan Wang [b], Andrew Beddows [j], Frank Kelly [j], Ben Barratt [j], Raphael E Arku [b,*]

[a] Department of Epidemiology and Biostatistics, School of Public Health, Imperial College London, London, UK
[b] Department of Environmental Health Sciences, School of Public Health and Health Sciences, University of Massachusetts, Amherst, USA
[c] School of Population and Public Health, The University of British Columbia, Vancouver, Canada
[d] MRC-PHE Center for Environment and Health, Imperial College London, London, UK
[e] Institute for Health and Social Policy, McGill University, Montreal, Canada
[f] Department of Epidemiology, Biostatistics, and Occupational Health, McGill University, Montreal, Canada
[g] Department of Physics, University of Ghana, Legon, Ghana
[h] Harvard T.H. Chan School of Public Health, Boston, MA, USA
[i] Department of Geography and Resource Development, University of Ghana, Legon, Ghana
[j] Department of Analytical, Environmental and Forensic Sciences, King's College London, London, UK

[*]Corresponding author:
Raphael E Arku
School of Public Health and Health Sciences
University of Massachusetts
Amherst, MA, USA
E-mail: rarku@umass.edu

Word count: 4121 words (excluding title page, abstract, references, figures and tables)

For peer review only

ABSTRACT

Introduction: Air and noise pollution are emerging environmental health hazards in African cities, with potentially complex spatial and temporal patterns. Limited local data is a barrier to the formulation and evaluation of policies to reduce air and noise pollution.

Methods and analysis: We designed a year-long measurement campaign to characterize air and noise pollution and their sources at high-resolution within the Greater Accra Metropolitan Area, Ghana. We will deploy low-cost, low-power, lightweight monitoring devices that are robust, socially unobtrusive, and able to function in the SSA climate. Our design utilizes a combination of fixed (year-long, n = 10) and rotating (week-long, n = ~130) sites, selected to represent a range of land uses and source influences (e.g. background, road-traffic, commercial, industrial, and residential areas, and various neighbourhood socioeconomic classes). We will collect data on fine particulate matter ($PM_{2.5}$), nitrogen oxides (NO_x), weather variables, sound (noise levels and audio), and street-level time-lapse images. We will use state-of-the-art methods, including spatial statistics, deep/machine learning, and processed-based emissions modelling, to capture highly resolved temporal and spatial variations in pollution levels across Accra and to identify their potential sources. This protocol can serve as a prototype for pollution monitoring in other rapidly growing Sub-Saharan African cities.

Ethics and dissemination: This environmental study was deemed exempt from full ethics review at Imperial College London and the University of Massachusetts Amherst; it was approved by the University of Ghana Ethics Committee. This protocol is designed to be implementable in Sub-Saharan African cities to map environmental pollution to inform urban planning decisions to reduce health harming exposures to air and noise pollution. It will be disseminated through local stakeholder engagement (public and private sectors), peer-reviewed publications, contribution to policy documents, media, and conference presentations.

Key words: Air pollution, noise pollution, environmental monitoring, environmental modelling, machine learning, urban health, health inequality, sub-Saharan Africa

For peer review only

STRENGTHS AND LIMITATIONS OF THE STUDY

- Our study is the largest air and noise pollution measurement campaign conducted in a major SSA city and serves as a prototype for data-poor SSA cities.
- The study relies on new sensor technologies to generate rich datasets on air and noise pollution levels along with street-level imagery and sound types that help identify sources across over 140 locations.
- Data from a combination of fixed (1 year) and rotating (7 day) monitoring sites representing various land-use types will allow for an assessment of both the temporal and spatial variability of pollution.
- While our study makes use of next-generation low-cost technologies, significant need for human resources is required for site identification and preparation, equipment deployment and maintenance, and data download and management.

For peer review only

INTRODUCTION

Sub-Saharan Africa (SSA) is the world's fastest urbanising region, with the number of urban dwellers having increased by over 400% since 1980 [1]. Urban growth in SSA has been largely unplanned especially in relation to housing, transport and energy. As a result, air and noise pollution are increasingly a public health concern for SSA urban residents [2–4]. For example, estimates from global models suggest that ambient fine particulate matter (Particulate Matter with diameter <2.5 micrometers ($PM_{2.5}$)) in SSA is well above levels in high-income North America and Western Europe [3,5]. The data from the few available measurement studies show that only about 10% of cities in SSA are meeting the WHO annual average Air Quality Guideline of 10 μg/m^3 [5,6]. Furthermore, the estimated annual number of deaths in some major SSA cities from $PM_{2.5}$ pollution in 2016 was as high as 5,640 to 520 in Johannesburg (South Africa), Lagos (Nigeria), Kinshasa (Democratic Republic of Congo), Dar es Salaam (Tanzania), Accra (Ghana), and Nairobi (Kenya) [7]. While such global estimates and the limited measurement data provide a broad view of air pollution, they do not capture the spatial variability and within-city disparities, nor do they provide information on sources [8–10]. Those within-city differences are important determinants of pollution related health inequalities. There are even less data on noise levels, and none on its health burden, and the limited data show much higher levels compared to cities in high-income countries [11–16], which may be associated with hearing loss, sleep disturbance, impaired cognitive function, and cardiovascular disease [17–19].

Air and noise pollution in SSA have a complex mix of local and regional sources: these include informal industries, transportation predominantly from old imported vehicles for commercial and private use, biomass use for household and commercial activities, household trash burning, re-suspended dust from unpaved roads, dust from regional dust storms, and noise from road-traffic, small road-side businesses, and religious practices, to name a few [4,8,10,20,21]. These sources

For peer review only

influence the pollutant mixture (e.g. PM, Nitrogen Oxides (NO/NO_2)) and the type of urban sounds, resulting in variation in spatial patterns and potentially differential impacts on health. Carefully designed measurements using low-cost robust sensors present an opportunity to provide data on air and noise pollution levels, variations, and sources, to inform and evaluate effectiveness of policies in SSA.

Motivated in part by detailed air pollution data from four neighborhoods in the city core, Accra, Ghana's largest city, in 2018 announced initiatives to reduce air pollution [22]; whereas noise is currently making headlines in both local and international media [23–25]. Our goal is to leverage advancement in sensor technology, modeling and image processing to design a measurement campaign combined with machine learning, statistical, and process-based modelling to characterize highly-resolved space-time variability of air and noise pollution, and their sources in the Greater Accra Metropolitan Area (GAMA). This work is nested within the larger multi-country and multi-city "Pathways to Equitable Healthy Cities" Study (http://equitablehealthycities.org/), which aims to identify and inform equitable and healthy urban development and revitalization pathways in six cities on four continents.

This paper details the protocol being used to collect and analyze pollution data in high resolution and provides practical guideline in a rapidly growing SSA metropolitan area. As one of the few studies of air and noise pollution at fine spatial resolution in an SSA city, this paper and the data to be generated make three main contributions. First, to develop and implement a data-rich measurement campaign on air and noise pollution in the GAMA that can provide spatially and temporally graded data. Second, to present a measurement protocol that can be readily adapted to other SSA cities. Third, to describe how the data will be utilized to fit and/ or validate geostatistical, machine learning, and process-based dispersion models that can predict pollution levels at high-spatial and temporal resolution and simulate and evaluate different policy scenarios on air quality in Accra.

For peer review only

METHODS AND ANLAYSIS

Study location and timeline

Our measurement campaign is focussed on the GAMA, which covers about 1500 km^2, and consists of multiple metropolis and municipalities, with Accra Metropolitan Area (AMA) at its core (Figure 1). Accra lies in the dry equatorial climate zone with rainy (May-September) and dry Harmattan seasons characterized by dusty north-easterly trade winds from the Sahara Desert. The elevation of GAMA is near sea level. Monthly average temperatures range from 27 to 32° C with average daily humidity of 79% [26]. As Ghana's capital and largest city, Accra has become one of SSA's hubs for business, technology, communications, and education. However, there remain large inequalities in housing and possibly exposure to environmental health risks [9,27–29].

We scheduled a one-year field measurement campaign to cover the rainy and Harmattan periods. Measurements began with a 3-week long pilot campaign in April 2019 and will continue until May 2020 (Figure 2).

Measurement campaign design

To capture the temporal (daily, weekly, seasonal) and spatial variations in both pollution and its sources across the entire study area, we are using a combination of 'fixed' and 'rotating' monitoring sites. The sites represent a blend of features such as background (e.g., low traffic and green space), low vs. high road-traffic, sparsely vs. densely built-up areas, poor vs. affluent, and established vs. emerging neighborhoods.

For peer review only

Ten fixed sites have been installed and will operate continuously all year long; the sites were purposefully selected based on the above criteria related to population density, road-traffic and road-networks, and on neighborhood socio-economic status and biomass fuel use based on national census data [30]. The sites included four locations used in an earlier air pollution study in the AMA. Additional provisions have been made to co-locate with two World Bank sponsored regulatory monitoring sites [27].

To capture spatial patterns of pollution while maximizing a finite number of sensor packages, we also operate sites that rotate weekly in order to capture the spatial variation in pollution levels and sources but also the temporal variation within and between days. In each measurement week, samples are collected at five new locations that continuously monitor for 7 days. By the end of the study, ~130 unique locations will be monitored for one week across the GAMA.

In selecting the rotating site locations, we used a stratified random sampling approach. The GAMA area was stratified by a 20m x 20m landcover dataset with four classes: low-density residential, high-density residential, commercial and business areas, and 'other' areas (e.g. parks, forest, agricultural areas) [31]. Sampling sites were randomly selected within strata without replacement with higher probability of selecting inside the inner-city core AMA (where most of the population lives). Prior to measurement, the field team conducts on-site visits to identify the appropriate sites at, or as close as possible to, the computer-generated "ideal" locations. When a computer-generated location is deemed unsuitable or in a restricted area (e.g. military barracks), a nearest suitable spot to the ideal location is identified as a replacement.

Measurement methods and equipment

We systematically selected and are employing low-cost, low-power, and lightweight monitors that are robust and able to function in an environment characterized by high temperatures and humidity,

For peer review only - http://bmjopen.bmj.com/site/about/guidelines.xhtml

rain and dust storms, and with limited and intermittent electricity supply from the grid, and at the same time are socially unobtrusive (Table 1, Figure 3).

Table 1. Features, dimensions, and prices of the monitors/ sensors

Monitor	Cost per unit (USD $)	Weight (g)	Dimensions (cm)	Battery/ power requirements	Memory requirements	Recording/ measurement interval	Measured parameters
*Ultrasonic Personal Aerosol Sampler (UPAS)	1200	230	12.8x7.0x3.3	Internal chargeable battery*	Micro SD	7 days	$PM_{2.5}$ integrated ($\mu g/m^3$)
*ZeFan continuous $PM_{2.5}$ monitor	70	150	10.6x6.3x2.6	Internal chargeable battery*	Internal memory (USB connection)	1 minute	$PM_{2.5}$ continuous ($\mu g/m^3$)
†Ogawa Nitrogen Dioxide (NO_2/ NOx) sampler	85	60	8.0x4.0x3.0	NA	NA	7 days	NO_2 (ppb) integrated; NOx (ppb) integrated
Noise Sentry sound level meter	306	100	7.6x3.9 x5.9	Internal chargeable battery	Internal memory (USB connection)	1 minute	Noise levels (dBA)
AudioMoth audio recorder	70	95	6.2x5.0x2.2	AA batteries	Micro SD	10 seconds every 10 minutes	Audio (.WAV file)
Kestrel weather meter	310	120	12.7x4.5x2.8	AA batteries	Internal memory (USB connection)	1 minute	Temperature; relative humidity; wind speed; wind direction
Moultrie camera trap	150	500	13.1x8.1x6.6	AA batteries	SD	5 minutes	Time-lapse imagery (.jpeg file)

dBA: Decibels A-weighted; $PM_{2.5}$: Particulate matter with aerodynamic diameter less than 2.5 micrometers; ppb: parts per billion

*UPAS and Zefan battery life can be extended using an external power bank. We used the always-on battery pack from Voltaic Systems (www.voltaicsystems.com).

†NO_2/ NOx: Nitrogen Dioxide/Oxides (price includes clip, screens, plastic re-sealable pouch and reusable airtight storage and shipping vial

Air pollution monitors

Integrated $PM_{2.5}$: The Ultrasonic Personal Aerosol Sampler (UPAS) [32] from Access Sensor Technologies (Fort Collins, USA) (UPAS) is a time-integrated $PM_{2.5}$ monitor and has a quiet solid-state miniature piezoelectric pump for drawing air through a customized cyclone onto a 37mm diameter filter media contained in barcoded cartridges within the device. With a mass flow sensor and controller, UPAS provides a steady flow rate over time. A mobile app makes UPAS easily programmable to collect samples at varying duty cycles. The UPAS devices are being operated at 1

For peer review only

liter per minute (lpm) at 50% to avoid overloading the weekly-integrated filters. The UPAS has been evaluated in laboratory and field settings against a federal reference monitor (URG-2000-30EGN-A; URG Corp., USA), personal environmental monitor (PEM 761‐203; SKC, Inc., USA) and Harvard Impactor, respectively and has proven valid for ambient, household, and personal monitoring in a typical tropical climate as our study [32–34].

Continuous $PM_{2.5}$: The ZeFan continuous monitor (http://www.zfznkj.com/) is a portable direct-reading $PM_{2.5}$ monitor that is based on light scattering technique [35]. ZeFan uses the Plantower sensor (model PMS7003) which has been validated against TEOM 1400a analyser and tested for durations ranging from 6 months to a year in various environmental conditions [35,36]. Prior to field deployment, we tested minute-by-minute monitor-monitor precision by running 15 monitors alongside each other over a 24-hour period at the University of Ghana, Legon campus with average relative humidity (RH) (~ 78%) and temperature (29 °C) representative of the city, and the measurements had good agreement (Figure 4). Mid-campaign precision test will be conducted and compared with the baseline data. Since light-scattering techniques only infer PM mass from detecting particle number concentrations and are impacted by weather conditions (i.e. RH and temperature), their estimates of mass concentration are inexact. Thus, we will co-locate the ZeFan with U.S. federal equivalent continuous monitor (Teledyne Model T640x) at two sites in Accra for a week in each season (dry vs. rain), up to four times a year and first adjust the minute-by-minute PM records for impact of RH and then their average against the co-located integrated $PM_{2.5}$ concentrations from UPAS.

Nitrogen Oxides (NO_x/NO_2): The Ogawa Passive Sampler (https://ogawausa.com) is being used to measure NO_x and NO_2, which are inorganic gas indicators of traffic related air pollution [37] and widely used in measurements to support land use regression modeling [38]. The sampler is easy to deploy, reusable, and does not require electricity, thus making it a cost-effective option in SSA settings. The sampler consists of two chambers with double sided diffusion that can concurrently

For peer review only

capture NO_x and NO_2 concentrations on collection pads pre-coated with 2-phenyl-4,4,5,5-tetramethylimidazoline-1-oxyl-3-oxide and triethanolamine, respectively. The samplers are covered by an opaque plastic container which serves as weather shield.

Sound monitors

Noise levels: The Type II Noise Sentry Sound Level Meter (SLM) datalogger (NSRT_mk3) from Convergence Instruments, Canada is being used to measure environmental noise levels at 1-minute intervals. The Noise Sentry is a relatively low-cost SLM for capturing and constructing common metrics of environmental noise with multiple weighting curves (A, C, Z). It is small and rugged, built to withstand temperatures in the range of -20°C to 60°C, and protected against water and dust. Previous studies have used the Noise Sentry SLM in diverse settings [11,14]. Our pre-pilot tests of monitor-monitor precision using 20 monitors were high with only a 0.9 A-weighted decibel difference between the highest and lowest monitoring period means (range in mean 16-hr LAeq: 45.6 to 46.5 dBA). The monitor-monitor precision test was done in Accra and SLMs were exposed 16hrs to multiple sound environments similar to what we would expect during the full monitoring campaign. Our Type II Noise Sentry SLMs were also validated in a separate aircraft noise study conducted in San Francisco against a Type I industry standard instrument (DUO 01dB) [39], and the agreement was high (mean and median second by second difference between the instruments was -0.42 and -0.38 dBA, respectively).

Audio: The AudioMoth audio recorder is a low-cost, full spectrum, acoustic logger developed by Open Acoustic Devices (Oxford, UK) [40]. The AudioMoth will complement the sound level meter by recording audio which will be used to classify different types of sounds in an urban environment (e.g., birds vs cars). The AudioMoths are set to a sampling rate of 32 kHz in our study to capture the majority of sound in the audible range [41].

For peer review only

Weather monitors

The Kestrel 5500 weather meter (Nielsen-Kellerman Inc., Boothwyn, PA, USA) is being utilized to record weather variables every minute. The Kestrel is a hand-held environmental meter and considered tough and immune to the elements. It tracks several weather parameters, including temperature, relative humidity, and heat index. It was selected for its low power consumption, large memory capacity (>10,000 data points), and dust-and water-proof properties. Kestrel 5500 has been used in several studies in diverse settings [42,43]. According to factory specifications, the accuracy of the instrument is 0.5°C for temperature and 2% for relative humidity.

Time-lapse cameras

To characterize sources of pollution in space and time, we use weatherproof and rugged time-lapse camera (Moultrie-50 camera trap, PERDIX wildlife, UK). The cameras are programmed to capture images at 5-minute intervals throughout the sampling period, including at night using infrared technology. Depending on the location, one or two cameras are mounted to capture multiple frames of view of potential pollution sources such as cars and community cookstoves.

Integrated equipment monitoring box

To house the equipment, we built integrated field measurement boxes using weather protective Seahorse (SE-300) cases. The cases were designed and weather tested to securely house each piece of equipment along with battery packs inside a single compartment, and could be mounted on poles/trees of different sizes using ratchet straps. The cameras are mounted on the outside of the box with rotational multi-access brackets for ease of orientation. Additionally, soundproof foam was

For peer review only

placed in-between the air monitors and the SLM to mitigate internal sound that might be generated from the quiet air pollution monitor pumps. NOx/NO_2 passive samplers and the AudioMoth audio recorders are placed outside of the measurement boxes in their own smaller weather protective plastic cases.

Equipment deployment and data capture

The field team identifies potential sites at or near the computer-generated ideal locations. The team then approaches residents, owners, or managers, of property and explains the study rational and seeks approval to install equipment for a 1-year (fixed sites) or 7-day (rotating sites) period. The team carry signed letters containing description of the research and contact information of project investigators at the University of Ghana. The site is then prepared, and the equipment box is mounted on metal poles, in care of an established contact person, and out of reach of people passing by. Depending on the specific site, the poles are secured on flat rooftops/ balconies of one-story buildings or directly in the ground (Figure 5) about 4 meters high (±1m) [44] with no direct obstruction within 2 meters as is a common practice in ambient air pollution and environmental noise measurement. The cameras are mounted on the outside of the box and secured in metal cases.

After deployment, the field team completes a short form, documenting information about the site, including the presence or absence of visible pollution sources (e.g., road-side cooking), mitigation factors (e.g., trees) or other points of interest, such as schools, police stations, or hospitals. For the rotating sites, the team returns seven-days after initial set-up to retrieve the equipment for data download and cleaning in the laboratory. The clean equipment is then re-deployed 48-hrs later at five new locations. For the fixed sites, replacement monitors, replacement batteries, and memory cards are swapped on site so as not to have a disruption in monitoring. Although equipment are installed in clusters of five sites at a time for efficient access, it still takes about a full working day to scout and

For peer review only - http://bmjopen.bmj.com/site/about/guidelines.xhtml

secure five new rotating sites, and about half a day to set up/ take down a set of sites, with another half a day in the lab for data download/upload and equipment preparation for the next installation.

Logistics and training

Our local field team comprises of three recent graduates from the University of Ghana and a taxicab driver, all with technical training needed to manage the field operation. The team is given project specific training to understand the site selection criteria and to collect high quality data. Additionally, periodic field visits and regular phone calls by researchers are made to maintain high quality data. In each neighborhood or community, the team identifies and works with a community member to establish trust and facilitate entry into that community.

Data handling

Weekly data are downloaded, saved in triplicate onto two external hard drives, and a third copy uploaded to a sever at Imperial College via an encrypted laptop. For the integrated $PM_{2.5}$ filter samples, pre-labeled 0.2µm pore size 37mm barcoded Teflon membrane filters (https://mtlcorp.com/filters/) are used and weighed pre- and post-sampling using an MTL AH500 automated robotic scale (http://www.mtlcorp.com/#/filter-weighing/) maintained in a temperature and RH-controlled laboratory (23 ± 2 ^{o}C, 35 ± 2% RH) at the University of British Columbia. The filter labels are scanned, time-stamped, placed in individual carriers, and loaded into the input silos for 48 hours to equilibrate to laboratory conditions before weighing. System generated weighing reports (e.g. balance stability) for each filter are issued for quality control purposes. The pre-weighed filters are scanned and paired to and placed in labeled petri-dishes which are then sealed into individual packages. Each petri-dish has four labels used to match the filter to the cartridge, UPAS monitor, and field log sheet during field work. After sampling, the filters are matched to, and placed

For peer review only

back in, their corresponding petri-dishes and shipped to the laboratory for post-weights. Detailed information on the filter handling process can be found elsewhere [33]. An emerging low-cost image-based approach will be applied to the post-weighed filters to estimate optical reflectance as a measure of black carbon (BC) concentration [33], the mass related to light absorption due to the presence of carbonaceous species.

NO_x/NO_2 samples are handled according to the protocol from Ogawa [45]. After assembly in the laboratory, the loaded samplers contained in an airtight container are exposed only on site for the entire sampling week. After sampling, the above procedure is again followed, and samples refrigerated until the exposed pads are shipped in airtight shipping vials for laboratory analysis. The final sample concentrations are determined based on the ratio of the sample absorbance (measured by spectrometer) to the slope of a prepared standard curve. The full analytical method is publicly available [45].

Quality assurance and control

Equipment are cleaned and prepared in a secure laboratory at the University of Ghana. The UPAS mass flow sensor maintains a steady sampling flow rate over time by internal measuring changes in pressure drop across the filter media (32). But as part of our quality assurance process, the flow rates (1 lpm) of the UPAS monitors are checked with a TSI Mass Flowmeter (4000 Series) for possible flow drift prior to and immediately after each monitoring session. The SLMs are calibrated prior to each monitoring session with a CA114 sound calibrator at 94.0 dB ±0.3 dB and 1000Hz ±0.5% (Convergence Instruments, Canada).

To ensure that the air and noise monitors agree with each other throughout the full campaign (i.e., monitor-monitor agreement does not drift away through continued use), we are collecting duplicate

For peer review only

samples from co-located instruments at 20% of our rotating measurement sites. The duplicates from the Zefan real-time $PM_{2.5}$ monitors, the sound level meters, and the integrated filter-based $PM_{2.5}$ monitors, will be evaluated on a minute-by-minute, 24hr, and weekly level for agreement, respectively, over a 7-day monitoring period. We also collect 20% field blanks for the integrated $PM_{2.5}$ and NO_x/NO_2 samples. Blank $PM_{2.5}$ samples are prepared as regular samples in the clean lab, brought to the field, and deployed in the same way as the regular sample, but without the pump being turned on. NO_x/NO_2 blanks are brought to the field sites but not exposed to air in their sealed canisters. During analysis, information from the blank samples will be used to account for residual contamination from the laboratory work, transportation, and field handling processes, which in a past study in Accra was minimal [9]. To ensure the plantower sensors in the Zefan real-time $PM_{2.5}$ monitors are not degrading over time and losing sensitivity in measuring peaks due to dust depositing on the sensor, light source, and mirror, we will conduct mid-campaign monitor-monitor precision and sensitivity tests by simultaneously co-locating them with U.S. federal equivalent continuous monitor (Teledyne T640x) for hourly $PM_{2.5}$ comparison over a one week period in the dry and rainy seasons.

Modelling and analysis

The data from this measurement campaign will serve as inputs into a diverse suite of state-of-the-art statistical models and machine learning approaches to (i) predict pollution levels in high spatial and temporal resolution across the GAMA, (ii) identify sources of pollution, and (iii) simulate the impacts of policy scenarios on pollution levels. Below are brief descriptions of some of our planned modelling and analysis activities following data collection. Future results-based papers will describe the modelling approaches in greater detail.

High-resolution estimates of air and noise pollution in the GAMA

For peer review only

The increasing availability of geospatial datasets with land use characteristics [46], road network information [47], and points of interest (e.g., locations of schools and hospitals), supports the development of land use regression (LUR) models to predict pollution levels for urban areas [48,49]. To date, most applications of LUR models for air and noise pollution have been in high income countries [14,15,48], with an emerging number in Asian cities and a limited number in African cities [11,38,50,51]. To provide high spatiotemporal resolution maps of air and noise pollution in the GAMA, we will build multiple space-time LUR models using year-long data on $PM_{2.5}$, BC, NO_2, and NO_x concentrations, and noise pollution indicators (i.e., Equivalent Continuous Sound levels (LAeq), Intermittency Ratios (IR)). The combination of minute-by-minute data and its aggregates in hourly, daily, and weekly will allow modelling spatial and temporal variability at different scales and characterising within- and between-day variations. We will obtain predictor variables from publicly available sources (e.g. OpenStreetMap), government databases, and satellite imagery to collate data on transportation networks (e.g., road-classes, traffic density), land cover/ land use, points of interest (e.g., schools, hospitals), green and blue spaces, and other environmental variables, such as meteorological conditions from our installed weather stations. Appropriate data checks will be done to ensure that model assumptions are met along with cross validation methods to assess model performance in different parts of the city.

Identification of sources of air and noise pollution with imagery

In addition to the LUR models, we will glean insights into determinants of air and noise pollution (i.e., potential sources) in both space and time by applying novel machine learning approaches to our street-view time-lapse images collected at the measurement sites [52]. We will use "Object Detection" algorithms, which build on Convolution Neural Network (CNN) architecture to identify predefined object classes within an image with a rectangular box bounding their presence (Figure 6). We will modify pre-existing algorithms to include custom object classes specific to our study context such as

For peer review only

roadside cookstoves and street loudspeakers [53–56]. Specifically, a team of researchers from London and Ghana will label a subset of images (~1000) with these custom classes which will act as a training dataset. Applying such methods to the images can create a temporal catalogue of a diverse set of objects present each location, which could then be used to predict and model pollutant levels in both space and time.

Similarly, CNN models like the CityNet model can be applied to the audio data to classify different sound types and identify noise pollution sources. The development of CityNet and other sound classifiers highlight recent advancements in this field, however, the transferability of the available models (which have predominantly been trained on data from high-income cities [41,57]) to a setting such as Accra will have to be tested and understood before put to use.

Air pollution impacts of policy and urban planning

We plan to use deterministic process-based models of air pollution to estimate the air pollution impacts of policies and urban planning decisions in Accra. Process based models such as meteorological chemical transport and dispersion models [58–60] can provide quantitative estimates of the air pollution impacts of different policy scenarios by modifying sources according to the specific scenario. The deterministic relationships between the model's inputs and outputs will be used in conjunction with the measurement data to calibrate the highly uncertain SSA emissions input data. The remaining measurements will then be used to validate the model's outputs. Following validation, the model can be used for ongoing policy and urban planning scenario testing exercises for emissions reduction policies in Accra, and other SSA cities with similar source profiles.

ETHICS AND DISSEMINATION

For peer review only

This environmental study was deemed exempt from full ethics review at Imperial College London and the University of Massachusetts Amherst; it was approved by the University of Ghana Ethics Committee. While pollution sources (cars, roadside cookstoves and loud speakers, etc.) are the targets of our field cameras and audio recorders, bystanders in public places and their voices may sometimes be in the mix. Monitors are placed at a height (~ 4 meters and above) where faces are normally not recognizable in the images and conversations unintelligible in the audio. Further, the audio recorders record for only 10 seconds every 10 minutes and an image is taken once every five minutes. Extra precautions (e.g. blurring of faces in imagery) is taken to maintain privacy of bystanders.

Both public and private stakeholders and relevant civil society groups will be invited to annual research consortium meetings where preliminary and final results will be shared. This will enable policymakers to frame and understand impacts of current and future policy scenarios. Additionally, results will be presented at international conferences and also published in peer-reviewed journals. Further, we will also engage with civil society through blog posts and other social media platforms.

ACKNOWLEDGMENTS

We thank John Phillip Pearce for his role in the design and development of the integrated pollution monitoring equipment box. Thank you to the staff of the Physics Department at the University of Ghana for their assistance in setting up the laboratory.

For peer review only

FIGURE LEGENDS

Figure 1. The Greater Accra Metropolitan Area (GAMA) and locations of the fixed and rotating sites. The road network data is from OpenStreetMap combined with data from Ghana Geological Survey Department and the background land cover shapefile is from the World Bank (2014). The inset shows background maps of Africa and Ghana (ESRI), along with the GAMA boundary from Ghana Statistical Service. **High-density residential** indicates neighborhoods with small, crowded, irregular buildings and narrow unidentifiable unpaved roads such as in shantytowns and slums. **Low-density residential** indicates neighborhoods with small regular planned buildings and indicate formal residential areas. **Commercial/ business/ industrial** indicates neighborhoods with large buildings that can be used for commercial, industrial, office, or warehouse purposes. **Other** indicates areas with large spaces of vegetation (e.g., dense forest), barren land (e.g., sand, soil), or water bodies.

Figure 2. Timeline of measurement campaign. Weekly measurements consist of continuous ($PM_{2.5}$ air concentration, noise levels, meteorological conditions, audio, and imagery) and integrated ($PM_{2.5}$ and NO_x concentration) samples. We chose weekly integrated samples for $PM_{2.5}$ and NO_x for logistical reasons (cost and time) as well as lessons from a previous study that showed relatively high temporal correlation between daily measurements [9].

Figure 3. Images of environmental monitoring equipment.

Figure 4. Smoothed time series of minute-by-minute $PM_{2.5}$ from 15 co-located real-time Zefan monitors in Accra. The levels were neither corrected for RH or against integrated filter-based data.

Figure 5. Deployment of the pollution measurement equipment.

Figure 6. Illustration of how object detection models and street-level imagery can be combined from the Accra campaign data to identify potential correlates of air and noise pollution. Information recorded on the bottom of the images includes the date and time, camera name, and the ambient temperature.

For peer review only

REFERENCES

1 UN. World Urbanization Prospects. 2018. https://population.un.org/wup/Publications/Files/WUP2018-KeyFacts.pdf

2 Lall SV, Henderson JV, Venables A. *Africa's cities: opening doors to the world.* Washington: : The World Bank 2017. doi:10.1596/978-1-4648-1044-2

3 Katoto PDMC, Byamungu L, Brand AS, *et al.* Ambient air pollution and health in Sub-Saharan Africa: Current evidence, perspectives and a call to action. *Environ Res* 2019;**173**:174–88. doi:10.1016/j.envres.2019.03.029

4 Petkova EP, Jack DW, Volavka-Close NH, *et al.* Particulate matter pollution in African cities. *Air Qual Atmos Heal* 2013;**6**:603–14. doi:10.1007/s11869-013-0199-6

5 World Health Organization. Ambient air pollution: a global assessment of exposure and burden of disease. 2016. https://www.who.int/phe/publications/air-pollution-global-assessment/en/

6 World Health Organization. Annual mean ambient PM2.5 (ug/m3) - from measurements, 2018 update. 2018.

7 Anenberg SC, Achakulwisut P, Brauer M, *et al.* Particulate matter-attributable mortality and relationships with carbon dioxide in 250 urban areas worldwide. *Sci Rep* 2019;**9**:1–6. doi:10.1038/s41598-019-48057-9

8 Dionisio K, Rooney MS, Arku RE, *et al.* Within-neighborhood patterns and sources of particle pollution: mobile monitoring and geographic information system analysis in four communities in Accra, Ghana. *Environ Health Perspect* 2010;:607–13. doi:10.1289/ehp.0901365

9 Dionisio K, Arku RE, Hughes AF, *et al.* Air Pollution in Accra Neighborhoods: Spatial, Socioeconomic, and Temporal Patterns. *Environ Sci Technol* 2010;**44**:2270–6. doi:10.1021/es903276s

10 Rooney MS, Arku RE, Dionisio KL, *et al.* Spatial and temporal patterns of particulate matter sources and pollution in four communities in Accra, Ghana. *Sci Total Environ* 2012;**435–436**:107–14. doi:10.1016/j.scitotenv.2012.06.077

11 Sieber C, Ragettli MS, Brink M, *et al.* Land use regression modeling of outdoor noise exposure in informal settlements in Western Cape, South Africa. *Int J Environ Res Public Health* 2017;**14**. doi:10.3390/ijerph14101262

12 Essandoh PK, Armah FA. Determination of ambient noise Levels in the main commercial area of Cape Cost. *Res J Environ Earth Sci* 2011;**3**:637–44.

13 Baloye DO, Palamuleni LG. A comparative land use-based analysis of noise pollution levels in selected urban centers of Nigeria. *Int J Environ Res Public Health* 2015;**12**:12225–46. doi:10.3390/ijerph121012225

14 Ragettli MS, Goudreau S, Plante C, *et al.* Statistical modeling of the spatial variability of environmental noise levels in Montreal, Canada, using noise measurements and land use characteristics. *J Expo Sci Environ Epidemiol* 2016;**26**:597–605. doi:10.1038/jes.2015.82

15 Aguilera I, Foraster M, Basagaña X, *et al.* Application of land use regression modelling to assess the spatial distribution of road traffic noise in three European cities. *J Expo Sci Environ Epidemiol* 2015;**25**:97–105. doi:10.1038/jes.2014.61

16 Kheirbek I, Ito K, Neitzel R, *et al.* Spatial variation in environmental noise and air pollution in New York City. *J Urban Heal* 2014;**91**:415–31. doi:10.1007/s11524-013-9857-0

17 Perron S, Plante C, Ragettli MS, *et al.* Sleep disturbance from road traffic, railways, airplanes and from total environmental noise levels in montreal. *Int J Environ Res Public Health* 2016;**13**. doi:10.3390/ijerph13080809

18 Clark C, Paunovic K. WHO environmental noise guidelines for the european region: A systematic review on environmental noise and cognition. *Int J Environ Res Public Health* 2018;**15**. doi:10.3390/ijerph15020285

19 Basner M, Babisch W, Davis A, *et al.* Auditory and non-auditory effects of noise on health.

For peer review only

Lancet 2014;**383**:1325–32. doi:10.1016/S0140-6736(13)61613-X
20 Zakpala RN, Armah FA, Sackey BM, *et al.* Night-Time Decibel Hell: Mapping Noise Exposure Zones and Individual Annoyance Ratings in an Urban Environment in Ghana. *Scientifica (Cairo)* 2014;**2014**:1–11. doi:10.1155/2014/892105
21 Zhou Z, Dionisio KL, Verissimo TG, *et al.* Chemical characterization and source apportionment of household fine particulate matter in rural, peri-urban, and urban West Africa. *Environ Sci Technol* 2014;**48**:1343–51. doi:10.1021/es404185m
22 Breathelife. BreathLife: Accra, Ghana. 2016.http://breathelife2030.org/breathelifecity/accra-ghana/ (accessed 12 Mar 2019).
23 Knott S, Gyamfi K. 'If you complain they see you as evil': Accra's religious noise problem. Guard. 2019.https://www.theguardian.com/cities/2019/mar/27/if-you-complain-they-see-you-as-evil-accras-religious-noise-problem (accessed 17 May 2019).
24 Bediako-Akoto RDO. Noise Pollution: A country at Risk. Dly. Graph. 2018;:1.https://www.graphic.com.gh/features/opinion/noise-pollution-a-country-at-risk.html
25 Kaledzi I. Ghana asks mosques to turn down the noise and use WhatsApp for call to prayer. 2018.https://www.dw.com/en/ghana-asks-mosques-to-turn-down-the-noise-and-use-whatsapp-for-call-to-prayer/a-43373007
26 Ghana Meteorological Agency. Ghana Meteorological Agency. http://www.meteo.gov.gh/website/index.php?option=com_content&view=article&id=87:regional-weather-greater-accra-region&catid=42:24-hour-forecast-for-ghana&Itemid=62 (accessed 6 Jul 2019).
27 Arku RE, Vallarino J, Dionisio KL, *et al.* Characterizing air pollution in two low-income neighborhoods in Accra, Ghana. *Sci Total Environ* 2008;**402**:217–31. doi:10.1016/j.scitotenv.2008.04.042
28 Boadi KO, Kuitunen M. Environmental and health impacts of household solid waste handling and disposal practices in Third World Cities: The case of the Accra Metropolitan Area, Ghana. *J Environ Health* 2005;**68**:32–6.
29 Songsore J, McGranahan G. Environment, wealth and health: towards an analysis of intra-urban differentials within the Greater Accra Metropolitan Area, Ghana. *Environ Urban* 1993;:10–30.
30 GSS. Ghana Population and Housing Census. 2010.http://www.statsghana.gov.gh/nada/index.php/catalog/51
31 World Bank. 2014 Land Cover Classification Of Accra, Ghana. 2014. https://datacatalog.worldbank.org/dataset/c-2014-land-cover-classification-accra-ghana
32 Volckens J, Quinn C, Leith D, *et al.* Development and evaluation of an ultrasonic personal aerosol sampler. *Indoor Air* 2017;**27**:409–16. doi:10.1111/ina.12318
33 Arku RE, Birch A, Shupler M, *et al.* Characterizing exposure to household air pollution within the Prospective Urban Rural Epidemiology (PURE) study. *Environ Int* 2018;**114**:307–17. doi:10.1016/j.envint.2018.02.033
34 Pillarisetti A, Carter E, Rajkumar S, *et al.* Measuring personal exposure to fine particulate matter (PM 2.5) among rural Honduran women: A field evaluation of the Ultrasonic Personal Aerosol Sampler (UPAS). *Environ Int* 2019;**123**:50–3. doi:10.1016/j.envint.2018.11.014
35 Bulot FMJ, Johnston SJ, Basford PJ, *et al.* Long-term field comparison of the performances of multiple low-cost particulate matter sensors in an urban area. *Sci Rep* 2019;:1–16. doi:10.5281/ZENODO.2531601
36 Malings C, Tanzer R, Hauryliuk A, *et al.* Fine particle mass monitoring with low-cost sensors: Corrections and long-term performance evaluation. *Aerosol Sci Technol* 2019;**0**:1–15. doi:10.1080/02786826.2019.1623863
37 Sather M, Slonecker T, Mathew J, *et al.* Evaluation of ogawa passive sampling devices as an alternative measurement method for the nitrogen dioxide annual standard in El Paso, Texas. *Environ Monit Assess* 2007;**124**:211–21.
38 He B, Heal MR, Reis S. Land-use regression modelling of intra-urban air pollution variation

For peer review only

in China: Current status and future needs. *Atmosphere (Basel)* 2018;**9**:1–19. doi:10.3390/atmos9040134
39 Rindleisch TC. A comparative evaluation of two sound level meters. 2018. doi:10.13140/RG.2.2.26395.31520
40 Hill AP, Prince P, Piña Covarrubias E, *et al.* AudioMoth: Evaluation of a smart open acoustic device for monitoring biodiversity and the environment. *Methods Ecol Evol* 2018;**9**:1199–211. doi:10.1111/2041-210X.12955
41 Fairbrass AJ, Firman M, Williams C, *et al.* CityNet—Deep learning tools for urban ecoacoustic assessment. Methods Ecol. Evol. 2018. doi:10.1111/2041-210X.13114
42 Xu M, Hong B, Jiang R, *et al.* Outdoor thermal comfort of shaded spaces in an urban park in the cold region of China. *Build Environ* 2019;**155**:408–20. doi:10.1016/j.buildenv.2019.03.049
43 Morguí J-A, Gacia E, Grossi C, *et al.* Atmospheric Carbon Dioxide variability at Aigüestortes, Central Pyrenees, Spain. *Reg Environ Chang* 2018;**19**:313–24. doi:10.1007/s10113-018-1443-2
44 Council of the European Union. Directive of The European Parliament and of The Council of 25 June 2002 relating to the assessment and management of environmental noise. 2002. https://publications.europa.eu/en/publication-detail/-/publication/27d1a64e-08f0-4665-a258-96f16c7af072/language-en
45 Ogawa. NO, NO2, NOx and SO2 sampling protocol using the Ogawa sampler. 2006. http://ogawausa.com/wp-content/uploads/2017/11/prono-noxno2so206_206_1117.pdf
46 Larkin A, Geddes JA, Martin R V., *et al.* Global land use regression model for nitrogen dioxide air pollution. *Environ Sci Technol* 2017;**51**:6957–64. doi:10.1021/acs.est.7b01148
47 Barrington-Leigh C, Millard-Ball A. The world's user-generated road map is more than 80% complete. *PLoS One* 2017;**12**:e0180698. doi:10.1371/journal.pone.0180698
48 Hoek G, Beelen R, de Hoogh K, *et al.* A review of land-use regression models to assess spatial variation of outdoor air pollution. *Atmos Environ* 2008;**42**:7561–78. doi:10.1016/j.atmosenv.2008.05.057
49 Khan J, Ketzel M, Kakosimos K, *et al.* Road traffic air and noise pollution exposure assessment – A review of tools and techniques. *Sci Total Environ* 2018;**634**:661–76. doi:10.1016/j.scitotenv.2018.03.374
50 Lee M, Brauer M, Wong P, *et al.* Land use regression modelling of air pollution in high density high rise cities: A case study in Hong Kong. *Sci Total Environ* 2017;**592**. doi:10.1016/j.scitotenv.2017.03.094
51 Saraswat A, Apte JS, Kandlikar M, *et al.* Spatiotemporal land use regression models of fine, ultrafine, and black carbon particulate matter in New Delhi, India. *Environ Sci Technol* 2013;**47**. doi:10.1021/es401489h
52 Weichenthal S, Hatzopoulou M, Brauer M. A picture tells a thousand…exposures: Opportunities and challenges of deep learning image analyses in exposure science and environmental epidemiology. *Environ Int* Published Online First: 2018. doi:10.1016/j.envint.2018.11.042
53 Redmon J, Divvala S, Girshick R, *et al.* You Only Look Once: Unified, Real-Time Object Detection. *Proc 2016 IEEE Conf Comput Vis Pattern Recognit (CVPR 2016)* 2015;:779–88. doi:10.1109/CVPR.2016.91
54 Lin TY, Maire M, Belongie S, *et al.* Microsoft COCO: Common objects in context. *Lect Notes Comput Sci (including Subser Lect Notes Artif Intell Lect Notes Bioinformatics)* 2014;**8693 LNCS**:740–55. doi:10.1007/978-3-319-10602-1_48
55 Liu W, Anguelov D, Erhan D, *et al.* SSD: Single shot multibox detector. *Lect Notes Comput Sci (including Subser Lect Notes Artif Intell Lect Notes Bioinformatics)* 2016;**9905 LNCS**:21–37. doi:10.1007/978-3-319-46448-0_2
56 Kuznetsova A, Rom H, Alldrin N, *et al.* The Open Images Dataset V4: Unified image classification, object detection, and visual relationship detection at scale. 2018;:1–

For peer review only

20.http://arxiv.org/abs/1811.00982
57 Salamon J, Bello JP. Deep Convolutional Neural Networks and Data Augmentation for Environmental Sound Classification. *IEEE Signal Process Lett* Published Online First: 2017. doi:10.1109/LSP.2017.2657381
58 Skamarock WC, Klemp JB. A time-split nonhydrostatic atmospheric model for weather research and forecasting applications. *J Comput Phys* 2008;**227**:3465–85.
59 Byun DW, Schere KL. Review of the governing equations, computational algorithms, and other components of the models-3 Community Multiscale Air Quality (CMAQ) modeling system. *Appl Mech Rev* 2006;**59**:51–77. doi:Doi 10.1115/1.2128636
60 Heist D, Isakov V, Perry S, *et al.* Estimating near-road pollutant dispersion: A model inter-comparison. *Transp Res Part D Transp Environ* 2013;**25**:93–105. doi:10.1016/j.trd.2013.09.003

Author contributions

All the authors contributed to this work and have taken part in the academic discussion for writing the study protocol, drafting the article and revising it. SNC, ASA, MB, ME, JB, MT, AH, JM, ST, JN, JV, SA, EA, BB, RA gave substantial contributions to conception and design and acquisition of data. SNC, ASA, MB, ME, MT, RN, EM, JW, AB, FK, RA gave substantial contributions to the analysis plan for data; SNC, ASA, MB, ME, JB, RA draft and revised the manuscript; and all authors reviewed the final version.

Funding

This work is supported by the Pathways to Equitable Healthy Cities grant from the Wellcome Trust [209376/Z/17/Z]. SC is supported by a Canadian Institutes for Health Research (CIHR) PhD scholarship as well as an Imperial College President's PhD scholarship. The content of this manuscript is solely the responsibility of the authors and does not necessarily represent the official views of the funders.

Ethics Approval

The study environmental was exempt from seeking ethics approval at Imperial College London and the University of Massachusetts-Amherst and was given ethical approval at the University of Ghana (ECH 149/ 18-19).

Competing interests

None

For peer review only

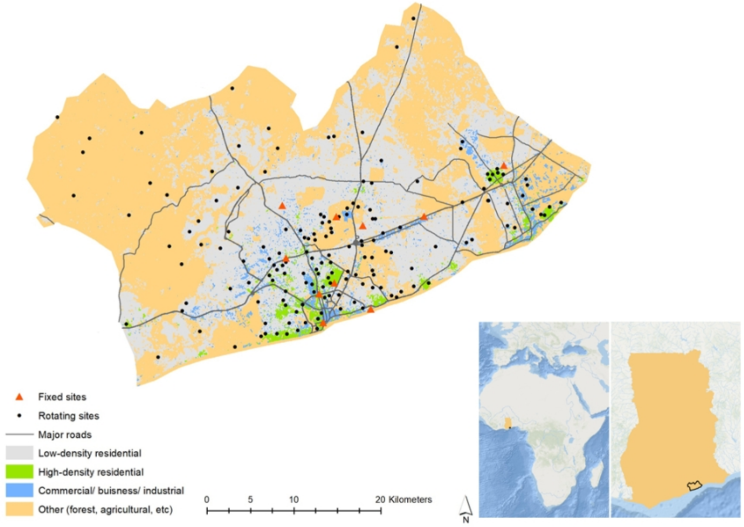

Figure 1. The Greater Accra Metropolitan Area (GAMA) and locations of the fixed and rotating sites. The road network data is from OpenStreetMap combined with data from Ghana Geological Survey Department and the background land cover shapefile is from the World Bank (2014). The inset shows background maps of Africa and Ghana (ESRI), along with the GAMA boundary from Ghana Statistical Service. High-density residential indicates neighborhoods with small, crowded, irregular buildings and narrow unidentifiable unpaved roads such as in shantytowns and slums. Low-density residential indicates neighborhoods with small regular planned buildings and indicate formal residential areas. Commercial/ business/ industrial indicates neighborhoods with large buildings that can be used for commercial, industrial, office, or warehouse purposes. Other indicates areas with large spaces of vegetation (e.g., dense forest), barren land (e.g., sand, soil), or water bodies.

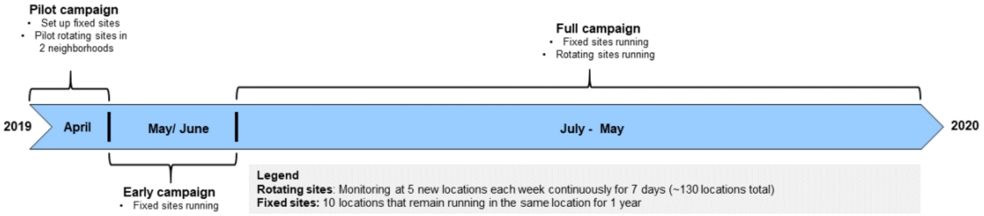

Figure 2. Timeline of measurement campaign. Weekly measurements consist of continuous (PM2.5 air concentration, noise levels, meteorological conditions, audio, and imagery) and integrated (PM2.5 and NOx concentration) samples. We chose weekly integrated samples for PM2.5 and NOx for logistical reasons (cost and time) as well as lessons from a previous study that showed relatively high temporal correlation between daily measurements.

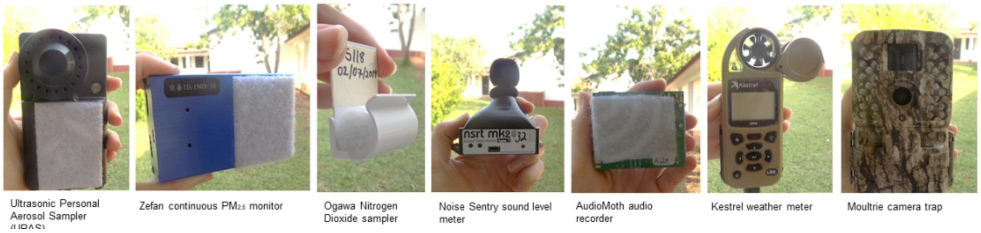

Figure 3. Images of environmental monitoring equipment.

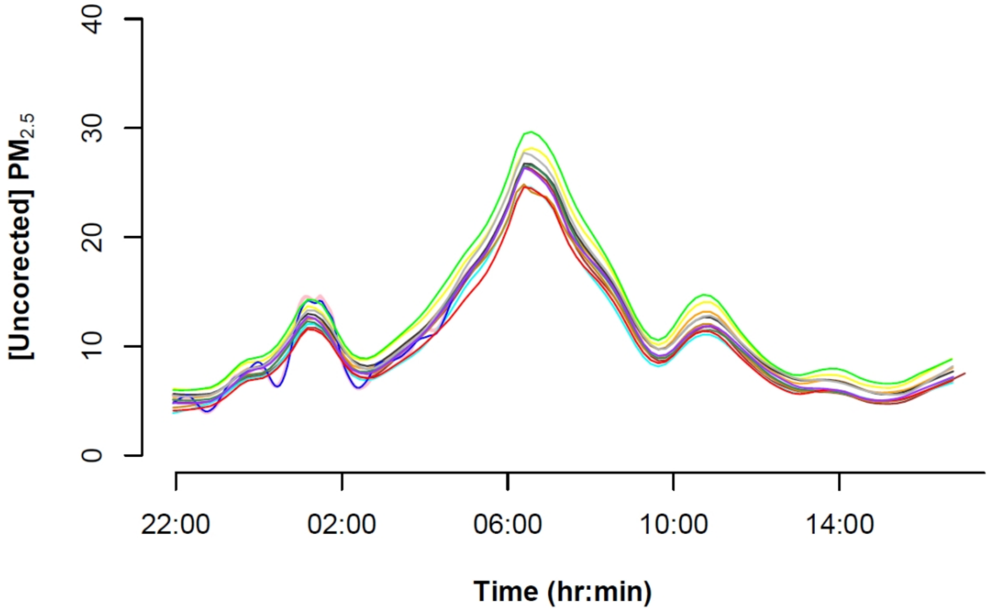

Figure 4. Smoothed time series of minute-by-minute PM2.5 from 15 co-located real-time Zefan monitors in Accra. The levels were neither corrected for RH or against integrated filter-based data.

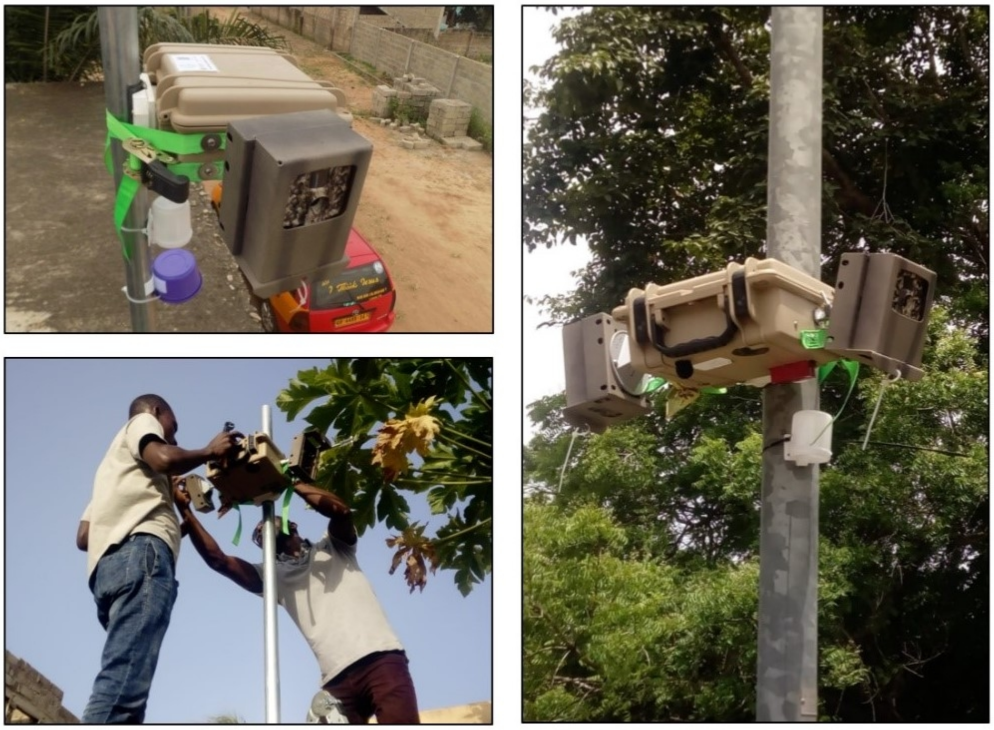

Figure 5. Deployment of the pollution measurement equipment.

265x195mm (96 x 96 DPI)

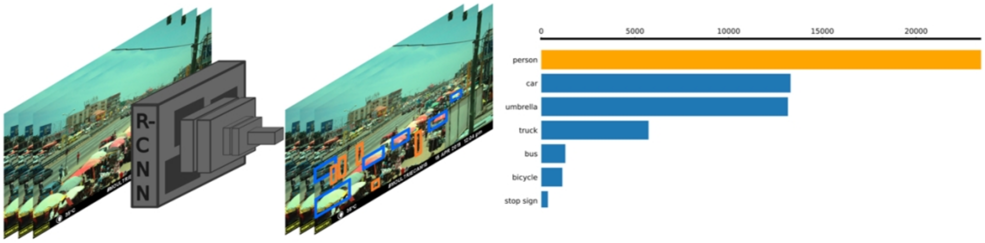

Figure 6. Illustration of how object detection models and street-level imagery can be combined from the Accra campaign data to identify potential correlates of air and noise pollution. Information recorded on the bottom of the images includes the date and time, camera name, and the ambient temperature.

BMJ Open

High-resolution spatiotemporal measurement of air and environmental noise pollution in sub-Saharan African cities: Pathways to Equitable Health Cities Study protocol for Accra, Ghana

Journal:	*BMJ Open*
Manuscript ID	bmjopen-2019-035798.R1
Article Type:	Protocol
Date Submitted by the Author:	06-Apr-2020

Complete List of Authors:	Clark, Sierra; Imperial College London, School of Public Health<br>Alli, Abosede; University of Massachusetts Amherst, Department of Environmental Health Sciences<br>Brauer, Michael; The University of British Columbia, School of Population and Public Health<br>Ezzati, Majid; Imperial College London, School of Public Health; Imperial College London, MRC-PHE Center for Environment and Health<br>Baumgartner, Jill ; McGill University, Institute for Health and Social Policy; McGill University, Department of Epidemiology, Biostatistics, and Occupational Health<br>Toledano, Mireille; Imperial College London, School of Public Health; Imperial College London, MRC-PHE Center for Environment and Health<br>Hughes, Allison; University of Ghana, Department of Physics<br>Nimo, James; University of Ghana, Department of Physics<br>Moses, Josephine; University of Ghana, Department of Physics<br>Terkpertey, Solomon; University of Ghana, Department of Physics<br>Vallarino, Jose; Harvard University, T.H. Chan School of Public Health<br>Agyei-Mensah, Samuel; University of Ghana, Department of Geography and Resource Development<br>Agyemang, Ernest; University of Ghana, Department of Geography and Resource Development<br>Nathvani, Ricky; Imperial College London, School of Public Health<br>Muller, Emily; Imperial College London, School of Public Health<br>Bennett, James; Imperial College London, School of Public Health<br>Wang, Jiayuan; University of Massachusetts Amherst, Department of Environmental Health Sciences<br>Beddows, Andrew; King's College London, Department of Analytical, Environmental and Forensic Sciences<br>Kelly, Frank; King's College London, Department of Analytical, Environmental and Forensic Sciences<br>Barratt, Benjamin; King's College London, Department of Analytical, Environmental and Forensic Sciences<br>Beevers, Sean; King's College London<br>Arku, Raphael; University of Massachusetts Amherst, Department of Environmental Health Sciences

**Primary Subject Heading**:	Research methods

Secondary Subject Heading:	Global health
Keywords:	STATISTICS & RESEARCH METHODS, EPIDEMIOLOGY, PUBLIC HEALTH

SCHOLARONE™
Manuscripts

I, the Submitting Author has the right to grant and does grant on behalf of all authors of the Work (as defined in the below author licence), an exclusive licence and/or a non-exclusive licence for contributions from authors who are: i) UK Crown employees; ii) where BMJ has agreed a CC-BY licence shall apply, and/or iii) in accordance with the terms applicable for US Federal Government officers or employees acting as part of their official duties; on a worldwide, perpetual, irrevocable, royalty-free basis to BMJ Publishing Group Ltd ("BMJ") its licensees and where the relevant Journal is co-owned by BMJ to the co-owners of the Journal, to publish the Work in this journal and any other BMJ products and to exploit all rights, as set out in our licence.

The Submitting Author accepts and understands that any supply made under these terms is made by BMJ to the Submitting Author unless you are acting as an employee on behalf of your employer or a postgraduate student of an affiliated institution which is paying any applicable article publishing charge ("APC") for Open Access articles. Where the Submitting Author wishes to make the Work available on an Open Access basis (and intends to pay the relevant APC), the terms of reuse of such Open Access shall be governed by a Creative Commons licence – details of these licences and which Creative Commons licence will apply to this Work are set out in our licence referred to above.

Other than as permitted in any relevant BMJ Author's Self Archiving Policies, I confirm this Work has not been accepted for publication elsewhere, is not being considered for publication elsewhere and does not duplicate material already published. I confirm all authors consent to publication of this Work and authorise the granting of this licence.

For peer review only

High-resolution spatiotemporal measurement of air and environmental noise pollution in sub-Saharan African cities: Pathways to Equitable Health Cities Study protocol for Accra, Ghana

Sierra N Clark [a], Abosede S Alli [b], Michael Brauer [c], Majid Ezzati [a,d,e,f], Jill Baumgartner [g,h], Mireille Toledano [a,d], Allison Hughes [i], James Nimo [i], Josephine Moses [i], Solomon Terkpertey [i], Jose Vallarino [j], Samuel Agyei-Mensah [k], Ernest Agyemang [k], Ricky Nathvani [a], Emily Muller [a], James Bennett [a,d], Jiayuan Wang [b], Andrew Beddows [d,l], Frank Kelly [d,l], Benjamin Barratt [d,l], Sean Beevers [d,l], Raphael E Arku [b,*]

[a] Department of Epidemiology and Biostatistics, School of Public Health, Imperial College London, London, UK
[b] Department of Environmental Health Sciences, School of Public Health and Health Sciences, University of Massachusetts, Amherst, USA
[c] School of Population and Public Health, The University of British Columbia, Vancouver, Canada
[d] MRC Center for Environment and Health, Imperial College London, London, UK
[e] Abdul Latif Jameel Institute for Disease and Emergency Analytics, Imperial College London, London, UK
[f] Regional Institute for Population Studies, University of Ghana, Legon, Ghana
[g] Institute for Health and Social Policy, McGill University, Montreal, Canada
[h] Department of Epidemiology, Biostatistics, and Occupational Health, McGill University, Montreal, Canada
[i] Department of Physics, University of Ghana, Legon, Ghana
[j] Harvard T.H. Chan School of Public Health, Boston, MA, USA
[k] Department of Geography and Resource Development, University of Ghana, Legon, Ghana
[l] Department of Analytical, Environmental and Forensic Sciences, King's College London, London, UK

[*]**Corresponding author:**
Raphael E Arku
School of Public Health and Health Sciences
University of Massachusetts
Amherst, MA, USA
E-mail: rarku@umass.edu

Word count: 4625

For peer review only

For peer review only - http://bmjopen.bmj.com/site/about/guidelines.xhtml

ABSTRACT

Introduction: Air and noise pollution are emerging environmental health hazards in African cities, with potentially complex spatial and temporal patterns. Limited local data is a barrier to the formulation and evaluation of policies to reduce air and noise pollution.

Methods and analysis: We designed a year-long measurement campaign to characterize air and noise pollution and their sources at high-resolution within the Greater Accra Metropolitan Area, Ghana. Our design utilizes a combination of fixed (year-long, n = 10) and rotating (week-long, n = ~130) sites, selected to represent a range of land uses and source influences (e.g. background, road-traffic, commercial, industrial, and residential areas, and various neighbourhood socioeconomic classes). We will collect data on fine particulate matter ($PM_{2.5}$), nitrogen oxides (NO_x), weather variables, sound (noise level and audio) along with street-level time-lapse images. We deploy low-cost, low-power, lightweight monitoring devices that are robust, socially unobtrusive, and able to function in the Sub-Saharan African (SSA) climate. We will use state-of-the-art methods, including spatial statistics, deep/machine learning, and processed-based emissions modelling, to capture highly resolved temporal and spatial variations in pollution levels across Accra and to identify their potential sources. This protocol can serve as a prototype for other SSA cities.

Ethics and dissemination: This environmental study was deemed exempt from full ethics review at Imperial College London and the University of Massachusetts Amherst; it was approved by the University of Ghana Ethics Committee. This protocol is designed to be implementable in SSA cities to map environmental pollution to inform urban planning decisions to reduce health harming exposures to air and noise pollution. It will be disseminated through local stakeholder engagement (public and private sectors), peer-reviewed publications, contribution to policy documents, media, and conference presentations.

For peer review only - http://bmjopen.bmj.com/site/about/guidelines.xhtml

Key words: Air pollution, noise pollution, sound classification, environmental monitoring, environmental modelling, machine learning, convolution neural network, urban health, health inequality, sub-Saharan Africa

For peer review only

For peer review only - http://bmjopen.bmj.com/site/about/guidelines.xhtml

STRENGTHS AND LIMITATIONS OF THE STUDY

- Our study is the largest air and noise pollution measurement campaign conducted in a major SSA city and serves as a prototype for other cities in SSA.
- The study relies on new sensor technologies to generate rich datasets on air and noise pollution along with imagery and audio recordings that help identify sources across ~ 140 locations.
- Data from a combination of fixed (1 year) and rotating (7 day) monitoring sites representing a diversity of areas will allow for an assessment of both the temporal and spatial variability of pollution.
- While our study makes use of next-generation low-cost technologies, significant need for human resources is required for site identification and preparation, equipment deployment and maintenance, and data download and management.

For peer review only

For peer review only - http://bmjopen.bmj.com/site/about/guidelines.xhtml

INTRODUCTION

Sub-Saharan Africa (SSA) is the world's fastest urbanising region, with the number of urban dwellers having increased by over 400% from 84 million in 1980 to an estimated urban population of ~450 million people in 2020 [1]. Urban growth in SSA has been largely unplanned especially in relation to housing, transport and energy. As a result, air and noise pollution are increasingly a public health concern for SSA urban residents [2–4]. For example, estimates from global models suggest that ambient fine particulate matter (Particulate Matter with diameter <2.5 micrometres ($PM_{2.5}$)) in SSA is well above levels in high-income North America and Western Europe [3,5]. The data from the few available measurement studies show that only about 10% of cities in SSA are meeting the World Health Organization (WHO) annual average Air Quality Guideline of 10 $\mu g/m^3$ [5,6]. While such global estimates and the limited measurement data provide a broad view of air pollution, they do not capture the spatial variability and within-city disparities, nor do they provide information on sources [7–9]. Those within-city differences are important determinants of pollution related health inequalities. There are even less data on noise pollution, and none on its health burden, and the limited data show much higher levels compared to cities in high-income countries [10–15], which may be associated with hearing loss, sleep disturbance, impaired cognitive function, and cardiovascular disease [16–18].

Air and noise pollution in SSA have a complex mix of local and regional sources: these include informal industries; transportation predominantly from old imported vehicles for commercial and private use; biomass use for household and commercial activities; household trash burning; re-suspended dust from unpaved roads; dust from regional dust storms; and noise from road-traffic, small road-side businesses, and religious practices, to name a few [4,7,9,19,20]. These sources influence the pollutant mixture (e.g. PM, Nitrogen Oxide (NO) and Nitrogen Dioxide (NO_2)) and the type of urban sounds, resulting in variation in spatial patterns and potentially differential impacts on health. Carefully designed measurements using low-cost robust sensors present an opportunity to

For peer review only - http://bmjopen.bmj.com/site/about/guidelines.xhtml

provide data on air and noise pollution levels, variations, and sources, to inform and evaluate the effectiveness of policies in SSA.

Motivated in part by earlier air pollution data from four neighbourhoods in the city core, Accra, Ghana's largest city, in 2018 announced initiatives to reduce air pollution [21]; whereas noise is currently making headlines in both local and international media [22–24]. Our goal is to leverage advancement in sensor technology, modelling and image processing to design a measurement campaign combined with machine learning, statistical, and process-based modelling to characterize highly resolved space-time variability of air and noise pollution, and their sources in the Greater Accra Metropolitan Area (GAMA). This work is nested within the larger multi-country and multi-city "Pathways to Equitable Healthy Cities" study (http://equitablehealthycities.org/), which aims to identify and inform equitable and healthy urban development and revitalization pathways in six cities on four continents.

This paper details the protocol being used to collect and analyse pollution data in high resolution and provides practical guideline in a rapidly growing SSA metropolitan area. As one of the few studies of air and noise pollution at fine spatial resolution in a SSA city, this paper and the data to be generated make three main contributions. First, to develop and implement a data-rich measurement campaign on air and noise pollution in the GAMA that can provide spatially and temporally graded data. Second, to present a measurement protocol that can be readily adapted to other SSA cities. Third, to describe how the data will be utilized to fit and/or validate geostatistical, machine learning, and physical dispersion models that can predict pollution levels at high-spatial and temporal resolution and simulate and evaluate different policy scenarios on air quality in Accra.

For peer review only

METHODS AND ANLAYSIS

Study location and timeline

Our measurement campaign is focussed on the GAMA, which covers about 1500 km^2, and consists of multiple metropolis and municipalities, with Accra Metropolitan Area (AMA) at its core (Figure 1). Accra lies in the dry equatorial climate zone with rainy (May-September) and dry Harmattan seasons characterized by dusty north-easterly trade winds from the Sahara Desert. The elevation of the GAMA is near sea level. Monthly average temperatures range from 27 to 32° C with average daily humidity of 79% [25]. As Ghana's capital and largest city, Accra has become one of SSA's hubs for business, technology, communications, and education. However, there remain large inequalities in housing and possibly exposure to environmental health risks [8,26–28].

We scheduled a one-year field measurement campaign to cover the rainy and Harmattan periods. Measurements began with a 3-week long pilot campaign in April 2019 and will continue until May 2020 (Figure 2).

Measurement campaign design

To capture the temporal (daily, weekly, seasonal) and spatial variations in both pollution and its sources across the entire study area, we are using a combination of 'fixed' and 'rotating' monitoring sites. The sites represent a blend of features such as background areas (e.g., low traffic and high green space), low vs. high road-traffic, sparsely vs. densely built-up areas, poor vs. affluent, and established vs. emerging neighbourhoods.

Ten fixed sites have been installed and will operate continuously all year long; the sites were purposefully selected based on the above criteria related to population density, road-traffic and road-networks, and on neighbourhood socio-economic status and biomass fuel use based on national

For peer review only - http://bmjopen.bmj.com/site/about/guidelines.xhtml

census data [29]. The sites included three locations used in an earlier air pollution study [7,26] in the Accra Metropolitan Area and additional provisions have been made to co-locate with World Bank sponsored regulatory monitoring sites and one at the U.S. Embassy.

To capture spatial patterns of pollution while maximizing a finite number of sensor packages, we also operate sites that rotate weekly in order to capture the spatial variation in pollution levels and sources as well as the temporal variation within and between days. In each measurement week, measurements are collected at four to five new locations that continuously monitor for seven days. By the end of the study ~130 unique locations will have been monitored for one week across the GAMA.

In selecting the rotating site locations, we used a stratified random sampling approach:

1. The study area (GAMA) was stratified by a land use grid (20m x 20m raster converted into a polygon shapefile) with four classes (medium/ low-density residential, high-density residential, commercial, business, and industrial areas, and 'other' areas (e.g. parks, forest, agricultural areas)) [30] and inside or outside the main Accra Metropolitan Area (AMA).
2. The computer then generated and returned the latitude/ longitude coordinates of a random sample of 130 target measurement site locations within strata.
3. Target measurement locations were first examined by overlaying point locations onto Google Maps and Google Earth to identify sites that were in restricted areas (e.g., military barracks). Sites in restricted areas were re-sampled to a nearest suitable spot that also fell within the same type of land use strata (n=~5 sites).
4. Using the coordinates of the target sampling locations, the field team then visit individual sites throughout the campaign to find measurement sites at or as close as possible to the target locations and also with the same land use characteristics.
5. When a site is deemed structurally sound for the field team to install equipment at (e.g., staircase to the roof) and can allow for the equipment to be installed at a target height, permission is requested from the site owner/ manager (more details on the logistics of field work are in sections below).

For peer review only - http://bmjopen.bmj.com/site/about/guidelines.xhtml

6. During the measurement campaign, we will actively review the balance between the number of actual measurement sites by land use strata as originally designed, and potentially sample additional sites to make up for unrepresentative site types.

Measurement methods and equipment

We systematically selected and are employing low-cost, low-power, and lightweight monitors that are robust and able to function in an environment characterized by high temperatures and humidity, rain and dust storms, and with limited and intermittent electricity supply from the grid, and at the same time are socially unobtrusive (Table 1, Figure 3).

Table 1. Features, dimensions, and prices of the monitors/ sensors

Monitor	Cost per unit (USD $)	Weight (g)	Dimensions (cm)	Battery/ power requirements	Memory requirements	Recording/ measurement interval	Measured parameters
*Ultrasonic Personal Aerosol Sampler (UPAS)	1200	230	12.8x7.0x3.3	Internal chargeable battery*	Micro SD	7 days	$PM_{2.5}$ integrated (µg/m^3)
*ZeFan continuous $PM_{2.5}$ monitor	70	150	10.6x6.3x2.6	Internal chargeable battery*	Internal memory (USB connection)	1 minute	$PM_{2.5}$ continuous (µg/m^3)
†Ogawa Nitrogen Dioxide (NO_2/ NOx) sampler	85	60	8.0x4.0x3.0	NA	NA	7 days	NO_2 (ppb) integrated; NOx (ppb) integrated
Noise Sentry sound level meter	306	100	7.6x3.9 x5.9	Internal chargeable battery	Internal memory (USB connection)	1 minute	Sound levels (dBA)
AudioMoth audio recorder	70	95	6.2x5.0x2.2	AA batteries	Micro SD	10 seconds every 10 minutes	Audio (.WAV file)
Kestrel weather meter	310	120	12.7x4.5x2.8	AA batteries	Internal memory (USB connection)	1 minute	Temperature; relative humidity; wind speed; wind direction
Moultrie camera trap	150	500	13.1x8.1x6.6	AA batteries	SD	5 minutes	Time-lapse imagery (.jpeg file)

dBA: A-weighted decibels; $PM_{2.5}$: Particulate matter with aerodynamic diameter less than 2.5 micrometres; ppb: parts per billion

*UPAS and Zefan battery life can be extended using an external power bank. We used the always-on battery pack from Voltaic Systems (www.voltaicsystems.com).

For peer review only - http://bmjopen.bmj.com/site/about/guidelines.xhtml

†NO_2/ NOx: Nitrogen Dioxide/Oxides (price includes clip, screens, plastic re-sealable pouch and reusable airtight storage and shipping vial

Air pollution monitors

Integrated $PM_{2.5}$: The Ultrasonic Personal Aerosol Sampler (UPAS) [31] from Access Sensor Technologies (Fort Collins, USA) (UPAS) is a time-integrated $PM_{2.5}$ monitor and has a quiet solid-state miniature piezoelectric pump for drawing air through a customized cyclone onto a 37mm diameter filter media contained in barcoded cartridges within the device. With a mass flow sensor and controller, UPAS provides a steady flow rate over time. A mobile app makes UPAS easily programmable to collect samples at varying duty cycles. The UPAS devices are being operated at 1 litre per minute (lpm) at 50% duty cycle to avoid overloading the weekly-integrated filters. The UPAS has been evaluated in laboratory and field settings against a federal reference monitor (URG ‐ 2000 ‐ 30EGN ‐ A; URG Corp., USA), personal environmental monitor (PEM 761 ‐ 203; SKC, Inc., USA) and Harvard Impactors, respectively and has proven valid for ambient, household, and personal monitoring in a typical tropical climate as our study [31–33].

Continuous $PM_{2.5}$: The ZeFan continuous monitor (http://www.zfznkj.com/) is a portable direct-reading $PM_{2.5}$ monitor that is based on light scattering technique [34]. ZeFan uses the Plantower sensor (model PMS7003) which has been validated against TEOM 1400a analyser and tested for durations ranging from 6 months to a year in various environmental conditions [34,35]. Prior to field deployment, we tested minute-by-minute monitor-monitor precision by running 15 monitors alongside each other over a 24-hour period at the University of Ghana, Legon campus with average relative humidity (RH) (~ 78%) and temperature (29 °C) representative of the city, and the measurements had good agreement (Figure 4). Since light-scattering techniques only infer PM mass from detecting particle number concentrations and are impacted by weather conditions (i.e. RH and temperature), their estimates of mass concentration are inexact. Thus, we will co-locate the ZeFan

For peer review only - http://bmjopen.bmj.com/site/about/guidelines.xhtml

with a U.S. federal equivalent continuous monitor Met One BAM 1020 at three sites, each with unique source influence in Accra for a week at the end of the campaign and adjust the minute-by-minute PM records for impact of RH and then their average against the co-located integrated $PM_{2.5}$ concentrations from the UPAS.

Nitrogen Oxides (NO_x/NO_2): The Ogawa Passive Sampler (https://ogawausa.com) is being used to measure NO_x and NO_2, which are inorganic gaseous indicators of traffic related air pollution [36]. The sampler is easy to deploy, reusable, and does not require electricity, thus making it a cost-effective option in SSA settings. The sampler consists of two chambers with double sided diffusion that can concurrently capture NO_x and NO_2 concentrations on collection pads pre-coated with 2-phenyl-4,4,5,5-tetramethylimidazoline-1-oxyl-3-oxide and triethanolamine, respectively. The samplers are covered by an opaque plastic container which serves as weather shield.

Sound monitors

Sound levels: The Type II Noise Sentry Sound Level Meter (SLM) datalogger (NSRT_mk3) from Convergence Instruments, Canada, is being used to measure sound levels at 1-minute integrating and logging intervals. The Noise Sentry is a relatively low-cost SLM for capturing and constructing common metrics of environmental noise pollution with multiple weighting curves (A, C, Z). It is small and rugged, built to withstand temperatures in the range of -20°C to 60°C, and protected against water and dust. Previous studies have used the Noise Sentry SLM in diverse settings [12,14]. Our pre-pilot tests of monitor-monitor precision showed good agreement (more details in supplementary information 1 (SI 1)). Our Type II Noise Sentry SLMs were also validated in a separate aircraft noise study conducted in San Francisco against a Type I industry standard instrument (DUO 01dB) [37], and the agreement was high (mean and median second by second difference between the instruments was -0.42 and -0.38 dBA, respectively).

For peer review only - http://bmjopen.bmj.com/site/about/guidelines.xhtml

Audio: The AudioMoth audio recorder is a low-cost, full spectrum, acoustic logger developed by Open Acoustic Devices (Oxford, UK) [38]. The AudioMoth will complement the sound level meter by recording audio which will be used to classify different types of sounds in an urban environment (e.g., animal vs vehicle sounds). The AudioMoths are set to a sampling rate of 32 kHz in our study to capture the majority of sound in the audible range [39].

Weather monitors

The Kestrel 5500 weather meter (Nielsen-Kellerman Inc., Boothwyn, PA, USA) is being utilized to record weather variables every minute. The Kestrel is a hand-held environmental meter and considered tough and immune to the elements. It tracks several weather parameters, including temperature, relative humidity, and heat index. It was selected for its low power consumption, large memory capacity (>10,000 data points), and dust-and water-proof properties. Kestrel 5500 has been used in several studies in diverse settings [40,41]. According to factory specifications, the accuracy of the instrument is 0.5°C for temperature and 2% for relative humidity.

Time-lapse cameras

To characterize sources of pollution in space and time, we use weatherproof and rugged time-lapse cameras (Moultrie-50 camera trap, PERDIX wildlife, UK). The cameras are programmed to capture images at 5-minute intervals throughout the sampling period, including at night using infrared technology. Depending on the location, one or two cameras are mounted to capture multiple frames of view of potential pollution sources in the street such as cars and community cookstoves.

Integrated equipment monitoring box

For peer review only - http://bmjopen.bmj.com/site/about/guidelines.xhtml

To house the equipment, we built integrated field measurement boxes using weather protective Seahorse (SE-300) cases. The cases were designed and weather tested to securely house each piece of equipment along with battery packs inside a single compartment, and could be mounted on poles of different sizes using ratchet straps. The cameras are mounted on the outside of the box with rotational multi-access brackets for ease of orientation. Additionally, soundproof foam was placed in-between the air monitors and the SLM to mitigate internal sound that might be generated from the quiet air pollution monitor pumps. NOx/NO_2 passive samplers and the audio recorders are placed outside of the measurement boxes in their own smaller weather protective plastic cases.

Equipment deployment and data capture

The field team identifies potential sites at or as near as possible to the computer-generated locations using direction from the saved locations on Google Maps. The team then approaches residents, owners, or managers, and explains the study rational and seeks approval to install equipment for a 1-year (fixed sites) or 7-day (rotating sites) period. The team carry signed letters containing description of the research and contact information of project investigators at the University of Ghana. The site is then prepared, and the equipment box is mounted on metal poles, in care of an established contact person, and out of direct reach of passers-by. Depending on the specific site, the poles are secured on flat rooftops/ balconies of one-story buildings or directly in the ground (Figure 5) about 4 meters high (±1m), as is a common practice in ambient air pollution and noise measurement [42], and also has no obstruction between the monitors and the sources of air and noise pollution. The cameras are mounted on the outside of the box and secured in metal cases.

After deployment, the field team completes a short form, documenting information about the site, including the presence or absence of visible pollution sources (e.g., road-side cooking), mitigation factors (e.g., trees) or other locations/ features of interest, such as road-side food sales, shopping centres, schools, or hospitals, etc. For the rotating sites, four to five locations are monitored each

For peer review only - http://bmjopen.bmj.com/site/about/guidelines.xhtml

week. Because of logistical and time constraints related to setting up each site, the team chooses sites that are within the same part of the city, but may have varying land use characteristics (e.g., mix of low and high-density residential locations). Monitors are retrieved seven days after initiating the measurements for data download and equipment cleaning in the field laboratory. The monitors are then re-deployed 48 hours later at a new set of locations in a different geographic area, with the aim of capturing potential microclimate and source-related differences between areas which likely impact pollution. For the fixed sites, replacement monitors, replacement batteries, and memory cards are swapped on site so as not to have a disruption in monitoring.

Logistics and training

Our local field team comprises of three recent graduates from the University of Ghana and a taxicab driver, all with technical training needed to manage the field operation. The team is given project specific training to understand the site selection criteria and to collect high quality data. Additionally, periodic field visits and regular phone calls by researchers are made to maintain high quality data. In each neighbourhood or community, the team identifies and works with a community member to establish trust and facilitate entry into that community.

Data handling

Weekly data are downloaded, saved in triplicate onto two external hard drives, and a third copy uploaded to a sever at Imperial College via an encrypted laptop. For the integrated $PM_{2.5}$ filter samples, pre-labelled 0.2µm pore size 37mm barcoded Teflon membrane filters (https://mtlcorp.com/filters/) are used and weighed pre- and post-sampling using an MTL AH500 automated robotic scale (http://www.mtlcorp.com/#/filter-weighing/) maintained in a temperature and RH-controlled laboratory (23 ± 2 ^{o}C, 35 ± 2% RH) at The University of British Columbia. The

For peer review only - http://bmjopen.bmj.com/site/about/guidelines.xhtml

filter labels are scanned, time-stamped, placed in individual carriers, and loaded into the input silos for 48 hours to equilibrate to laboratory conditions before weighing. System generated weighing reports (e.g. balance stability) for each filter are issued for quality control purposes. Samples are weighed thrice in both pre- and post-weighing and the average of the three measured masses is used for calculating concentrations. The pre-weighed filters are scanned and paired to and placed in labelled petri-dishes which are then sealed into individual packages. Each petri-dish has four labels used to match the filter to the cartridge, UPAS monitor, and field log sheet during field work. After sampling, the filters are matched to, and placed back in, their corresponding petri-dishes and shipped to the laboratory for post-weights. Detailed information on the filter handling process can be found elsewhere [32]. An emerging low-cost image-based approach will be applied to the post-weighed filters to estimate optical reflectance as a measure of black carbon (BC) concentration [43], the mass related to light absorption due to the presence of carbonaceous species.

NO_x/NO_2 samples are handled according to the protocol from Ogawa [44]. After assembly in the laboratory, the loaded samplers contained in an airtight container are exposed only on site for the entire sampling week. After sampling, the above procedure is again followed, and samples refrigerated until the exposed pads are shipped in airtight shipping vials for laboratory analysis. The final sample concentrations are determined based on the ratio of the sample absorbance (measured by spectrometer) to the slope of a prepared standard curve. The full analytical method is publicly available [44].

Quality assurance and control

Throughout the campaign, we will follow a set of procedures and protocols to uphold and assess the quality of the data being generated. We follow the principles that all procedures should be carefully planned, tested, and performed, the origin and life-course of all data must be traceable, and any deviations or irregularities must be recorded. Throughout all data collection, documentation of

For peer review only - http://bmjopen.bmj.com/site/about/guidelines.xhtml

sampling and conditions will be maintained in field notebooks. Furthermore, data collection logs will be digitized and backed up electronically on hard-drives and an online server, which will be checked on a daily basis for accuracy. The field team were given multiple weeks of project specific training prior to the pilot measurements. The team were taught specific protocols for equipment handling and cleaning, data inspection and cleaning, and equipment installation at measurement sites. The team were also given hardcopies of the protocols and, in addition to field visits by researchers, had constant remote access via phone/ web to project researchers throughout the campaign. In the supplementary information, we have included further information on our precision and accuracy testing, protocol for blank and duplicate collection, and data cleaning and inspecting procedures (SI 1).

For peer review only

Modelling and analysis

The data from this measurement campaign will be used to characterize the spatial and temporal patterns of air and noise pollution and serve as inputs into a diverse suite of state-of-the-art statistical, physical and machine learning models to (i) predict pollution levels in high spatial and temporal resolution across the GAMA, (ii) identify sources of pollution, and (iii) simulate the impacts of policy scenarios on air pollution levels. Below are brief descriptions of some of our planned modelling and analysis activities following data collection. Future results-based papers will describe the modelling approaches in greater detail.

Descriptive summaries of the spatial and temporal variations in air and noise pollution

We will provide summary statistics and visuals of the spatial and temporal patterns (within- and between-day, and seasonal) of air pollution ($PM_{2.5}$, NO_2) concentrations and average-based metrics of noise pollution such as $LAeq_{24hr}$, day-time (L_{day}), night-time (L_{night}), and day-evening-night weighted L_{den}. Additionally, we will include metrics which capture short-term and episodic sound

events such as the average maximum sound level and a novel metric that captures the percentage of event-based sound (the Intermittency Ratio (IR_{24hr}, IR_{day}, IR_{night})) [45].

High-resolution modelling of air and noise pollution in the GAMA

The increasing availability of geospatial datasets with land use characteristics [46], road network information [47], and locations of interest (e.g., locations of schools and hospitals) [48], supports the development of land use regression (LUR) models to predict pollution levels for urban areas [49,50]. To date, most applications of LUR models for air and noise pollution have been in high income countries [13,14,49], with an emerging number in Asian cities and a limited number in African cities [12,51–53]. To generate high resolution estimates of air and noise pollution in the GAMA, we will build LUR models with spatial and temporal predictor variables. The models will also include terms that allow for the capturing of systematic temporal patterns, e.g. random intercepts for hour of the day or month of the year, and terms that use pollution levels at fixed sites to remove weekly temporal changes. The models will use year-long data on $PM_{2.5}$, BC, NO_2, and NO_x concentrations, aggregated to weekly average concentrations, and sound level metrics aggregated hourly ($LAeq_{1hr}$) and daily (IR_{24hr}). The LAeq metric will be modelled hourly so that within-day patterns of sound variation can be captured in the model and then model predictions can be used to construct $LAeq_{24hr}$, L_{day}, L_{night}, and L_{den}. The specific temporal and spatial structures that are built into the models will be determined from the descriptive work.

We will obtain spatial/ location-based predictor variables from publicly available sources (e.g. OpenStreetMap), government databases, and satellite imagery to collate data on transportation networks (e.g., road-type), land cover/land use, points of interest (e.g., traffic lights, restaurants), and green and blue spaces. We also have temporal information on meteorological conditions (e.g., temperature, wind speed and direction, humidity) from local weather stations that co-located with 6 fixed-site environmental monitors. Appropriate data checks will be done to ensure that model

For peer review only - http://bmjopen.bmj.com/site/about/guidelines.xhtml

assumptions are met along with 10-fold hold-out cross validation methods to assess model performance in different parts of the city. Possible spatial autocorrelation in the data will be investigated by generating variogram plots of the raw data and the model residuals.

We will be reporting the model results in the form of estimates that represent annual average levels of $PM_{2.5}$, NO_2, NO_x, $LAeq_{24hr}$, L_{den} and IR_{24hr}. We will also provide maps that show estimates that are disaggregated by season (e.g. Harrmattan and non-Harrmattan for air pollution) and within day (e.g., day vs night).

Identification of sources of air and noise pollution with imagery and audio

We will glean insights into the determinants and correlates of air and noise pollution (i.e., potential sources) in both space and time by applying machine learning approaches, novel in the domain of pollution research, to our time-lapse images collected every five minutes at the ~ 140 measurement sites [54]. We will use Object Detection algorithms, implemented in a Convolution Neural Network architecture, to identify predefined object classes within an image with a rectangular box bounding their presence (Figure 6). We will modify pre-existing algorithms to include custom object classes specific to our study context such as roadside cookstoves and street loudspeakers, and as determined by the research team [55–58]. A sample set of pre-labelled images will be used to fine-tune a pre-trained object detection algorithm to detect the objects of relevance to this study. The algorithm will then be applied to all images collected during the campaign to produce a list of variables that can be included as independent variables in models estimating the association of air and noise pollution levels with the occurrence of these variables in high spatial-temporal resolution. This approach could be extended to potential future applications such as estimating traffic flows (segmented by vehicle type such as bicycles, cars and minivans whose average emissions vary) or to apply the model to new sources of street level imagery data to identify correlates of air and noise pollution at unmeasured locations across the city [54].

For peer review only - http://bmjopen.bmj.com/site/about/guidelines.xhtml

Similarly, machine learning models can be applied to the audio to classify different sound types and identify sound sources. Some models can predict over 500 different sound types/ sources (e.g., dog barking, ocean waves, car engine revving) and have been pre-trained on 2 million short audio clips [59,60]. The recent wave of development of these models highlight advancements in this field, however, the transferability of the available models (which have predominantly been trained on data from high-income cities and countries [39,59,61]) to a setting such as Accra will have to be tested and understood before put to use.

Air pollution impacts of policy and urban planning

We plan to use deterministic process-based models of air pollution to estimate the air pollution impacts of policies and urban planning decisions in Accra. Process based models such as meteorological chemical transport and dispersion models [62–64] can provide quantitative estimates of the air pollution impacts of different policy scenarios by modifying sources according to the specific scenario. After minimizing errors in meteorological inputs by nudging to ECMWF meteorological re-analysis data, the deterministic relationships between the model's emissions inputs and concentration outputs will be used in conjunction with the measurement data to calibrate the highly uncertain SSA emissions data. This relationship will be recreated using Gaussian process emulation [65] to simulate the millions of model runs required for a Bayesian Monte Carlo calibration [66] exercise, in which each run is weighted according to its output's agreement with the measurements. The same weights are applied to the corresponding emissions inputs, producing a distribution of emissions values, the modal value of which is taken as the calibrated input. Repeating this at multiple model time-steps averages the calibration over the values of the many other varying model inputs. The remaining measurements will then be used to validate the model's outputs, after it is re-run with the calibrated emissions. Following validation, the model (if appearing to perform well)

For peer review only - http://bmjopen.bmj.com/site/about/guidelines.xhtml

can be used for ongoing policy and urban planning scenario testing exercises for emissions reduction policies in Accra, and other SSA cities with similar source profiles.

PATIENT AND PUBLIC INVOLEMENT

No patients or members of the public were involved in this component of the study.

ETHICS AND DISSEMINATION

This environmental study was deemed exempt from full ethics review at Imperial College London and the University of Massachusetts Amherst; it was approved by the University of Ghana Ethics Committee. While pollution sources (cars, roadside cookstoves and loudspeakers, etc.) are the targets of our field camera and audio recorders, bystanders in public places and their voices may sometimes be in the mix. Monitors are placed at a height (~ 4 meters and above) where faces are normally not recognizable in the images and conversations unintelligible in the audio. Further, the audio recorders record for only 10 seconds every 10 minutes. Extra precautions (e.g. blurring of faces in imagery) is taken to maintain privacy of bystanders.

Both public and private stakeholders and relevant civil society groups will be invited to annual research consortium meetings where preliminary and final results will be shared. This will enable policymakers to frame and understand impacts of current and future policy scenarios. Additionally, results will be presented at international conferences and published in peer-reviewed journals. Further, we will also engage with civil society through blog posts and other social media platforms.

For peer review only - http://bmjopen.bmj.com/site/about/guidelines.xhtml

ACKNOWLEDGMENTS

We thank John Phillip Pearce for his role in the design and development of the integrated pollution monitoring equipment box. Thank you to the staff of the Physics Department at the University of Ghana for their assistance in setting up the laboratory.

For peer review only

For peer review only - http://bmjopen.bmj.com/site/about/guidelines.xhtml

FIGURE LEGENDS

Figure 1. The Greater Accra Metropolitan Area (GAMA) and locations of the fixed and computer-generated (sampled) rotating sites. The road network data is from OpenStreetMap and the background land cover shapefile is from the World Bank (2014). The inset shows background maps of Africa and Ghana (ESRI), along with the GAMA boundary from Ghana Statistical Service. **High-density residential** indicates neighborhoods with small, crowded, irregular buildings and narrow unidentifiable unpaved roads such as in shantytowns and slums. **Medium/ Low-density residential** indicates neighborhoods with small regular planned buildings and indicate formal residential areas. **Commercial/ business/ industrial** indicates neighborhoods with large buildings that can be used for commercial, industrial, office, or warehouse purposes. **Other** indicates areas with large spaces of vegetation (e.g., dense forest), barren land (e.g., sand, soil), or water bodies.

For peer review only

For peer review only - http://bmjopen.bmj.com/site/about/guidelines.xhtml

Figure 2. Timeline of measurement campaign. Weekly measurements consist of continuous ($PM_{2.5}$ air concentration, noise levels, meteorological conditions, audio, and imagery) and integrated ($PM_{2.5}$ and NO_x concentration) samples. We chose weekly integrated samples for $PM_{2.5}$ and NO_x for logistical reasons (cost and time) as well as lessons from a previous study that showed relatively high temporal correlation between daily measurements [8].

For peer review only

For peer review only - http://bmjopen.bmj.com/site/about/guidelines.xhtml

Figure 3. Images of environmental monitoring equipment.

For peer review only

For peer review only - http://bmjopen.bmj.com/site/about/guidelines.xhtml

Figure 4. Smoothed time series of minute-by-minute $PM_{2.5}$ from 15 co-located real-time Zefan monitors in Accra. The levels were neither corrected for relative humidity or against integrated filter-based data.

For peer review only

Figure 5. Deployment of the pollution measurement equipment.

For peer review only

For peer review only - http://bmjopen.bmj.com/site/about/guidelines.xhtml

Figure 6. Illustration of how object detection models and street-level imagery can be combined from the Accra campaign data to identify potential correlates of air and noise pollution in the imagery. Information recorded on the bottom of the images includes the date and time, camera name, and the ambient temperature.

For peer review only

For peer review only - http://bmjopen.bmj.com/site/about/guidelines.xhtml

REFERENCES

1 UN. World Urbanization Prospects. 2018. https://population.un.org/wup/Publications/Files/WUP2018-KeyFacts.pdf
2 Lall SV, Henderson JV, Venables A. *Africa's cities: opening doors to the world*. Washington: : The World Bank 2017. doi:10.1596/978-1-4648-1044-2
3 Katoto PDMC, Byamungu L, Brand AS, *et al.* Ambient air pollution and health in Sub-Saharan Africa: Current evidence, perspectives and a call to action. *Environ Res* 2019;**173**:174–88. doi:10.1016/j.envres.2019.03.029
4 Petkova EP, Jack DW, Volavka-Close NH, *et al.* Particulate matter pollution in African cities. *Air Qual Atmos Heal* 2013;**6**:603–14. doi:10.1007/s11869-013-0199-6
5 World Health Organization. Ambient air pollution: a global assessment of exposure and burden of disease. 2016. https://www.who.int/phe/publications/air-pollution-global-assessment/en/
6 World Health Organization. Annual mean ambient PM2.5 (ug/m3) - from measurements, 2018 update. 2018.
7 Dionisio K, Rooney MS, Arku RE, *et al.* Within-neighborhood patterns and sources of particle pollution: mobile monitoring and geographic information system analysis in four communities in Accra, Ghana. *Environ Health Perspect* 2010;:607–13. doi:10.1289/ehp.0901365
8 Dionisio K, Arku RE, Hughes AF, *et al.* Air Pollution in Accra Neighborhoods: Spatial, Socioeconomic, and Temporal Patterns. *Environ Sci Technol* 2010;**44**:2270–6. doi:10.1021/es903276s
9 Rooney MS, Arku RE, Dionisio KL, *et al.* Spatial and temporal patterns of particulate matter sources and pollution in four communities in Accra, Ghana. *Sci Total Environ* 2012;**435–436**:107–14. doi:10.1016/j.scitotenv.2012.06.077
10 Essandoh PK, Armah FA. Determination of ambient noise Levels in the main commercial area of Cape Cost. *Res J Environ Earth Sci* 2011;**3**:637–44.
11 Baloye DO, Palamuleni LG. A comparative land use-based analysis of noise pollution levels in selected urban centers of Nigeria. *Int J Environ Res Public Health* 2015;**12**:12225–46. doi:10.3390/ijerph121012225
12 Sieber C, Ragettli MS, Brink M, *et al.* Land use regression modeling of outdoor noise exposure in informal settlements in Western Cape, South Africa. *Int J Environ Res Public Health* 2017;**14**. doi:10.3390/ijerph14101262
13 Aguilera I, Foraster M, Basagaña X, *et al.* Application of land use regression modelling to assess the spatial distribution of road traffic noise in three European cities. *J Expo Sci Environ Epidemiol* 2015;**25**:97–105. doi:10.1038/jes.2014.61
14 Ragettli MS, Goudreau S, Plante C, *et al.* Statistical modeling of the spatial variability of environmental noise levels in Montreal, Canada, using noise measurements and land use characteristics. *J Expo Sci Environ Epidemiol* 2016;**26**:597–605. doi:10.1038/jes.2015.82
15 Kheirbek I, Ito K, Neitzel R, *et al.* Spatial variation in environmental noise and air pollution in New York City. *J Urban Heal* 2014;**91**:415–31. doi:10.1007/s11524-013-9857-0
16 Perron S, Plante C, Ragettli MS, *et al.* Sleep disturbance from road traffic, railways, airplanes and from total environmental noise levels in montreal. *Int J Environ Res Public Health* 2016;**13**. doi:10.3390/ijerph13080809
17 Clark C, Paunovic K. WHO environmental noise guidelines for the european region: A systematic review on environmental noise and cognition. *Int J Environ Res Public Health* 2018;**15**. doi:10.3390/ijerph15020285
18 Basner M, Babisch W, Davis A, *et al.* Auditory and non-auditory effects of noise on health. *Lancet* 2014;**383**:1325–32. doi:10.1016/S0140-6736(13)61613-X
19 Zakpala RN, Armah FA, Sackey BM, *et al.* Night-Time Decibel Hell: Mapping Noise Exposure Zones and Individual Annoyance Ratings in an Urban Environment in Ghana.

For peer review only - http://bmjopen.bmj.com/site/about/guidelines.xhtml

Scientifica (Cairo) 2014;**2014**:1–11. doi:10.1155/2014/892105
20 Zhou Z, Dionisio KL, Verissimo TG, *et al.* Chemical characterization and source apportionment of household fine particulate matter in rural, peri-urban, and urban West Africa. *Environ Sci Technol* 2014;**48**:1343–51. doi:10.1021/es404185m
21 Breathelife. BreathLife: Accra, Ghana. 2016.http://breathelife2030.org/breathelifecity/accra-ghana/ (accessed 12 Mar 2019).
22 Knott S, Gyamfi K. 'If you complain they see you as evil': Accra's religious noise problem. Guard. 2019.https://www.theguardian.com/cities/2019/mar/27/if-you-complain-they-see-you-as-evil-accras-religious-noise-problem (accessed 17 May 2019).
23 Bediako-Akoto RDO. Noise Pollution: A country at Risk. Dly. Graph. 2018;:1.https://www.graphic.com.gh/features/opinion/noise-pollution-a-country-at-risk.html
24 Kaledzi I. Ghana asks mosques to turn down the noise and use WhatsApp for call to prayer. 2018.https://www.dw.com/en/ghana-asks-mosques-to-turn-down-the-noise-and-use-whatsapp-for-call-to-prayer/a-43373007
25 Ghana Meteorological Agency. Ghana Meteorological Agency. http://www.meteo.gov.gh/website/index.php?option=com_content&view=article&id=87:regional-weather-greater-accra-region&catid=42:24-hour-forecast-for-ghana&Itemid=62 (accessed 6 Jul 2019).
26 Arku RE, Vallarino J, Dionisio KL, *et al.* Characterizing air pollution in two low-income neighborhoods in Accra, Ghana. *Sci Total Environ* 2008;**402**:217–31. doi:10.1016/j.scitotenv.2008.04.042
27 Boadi KO, Kuitunen M. Environmental and health impacts of household solid waste handling and disposal practices in Third World Cities: The case of the Accra Metropolitan Area, Ghana. *J Environ Health* 2005;**68**:32–6.
28 Songsore J, McGranahan G. Environment, wealth and health: towards an analysis of intra-urban differentials within the Greater Accra Metropolitan Area, Ghana. *Environ Urban* 1993;:10–30.
29 GSS. Ghana Population and Housing Census. 2010.http://www.statsghana.gov.gh/nada/index.php/catalog/51
30 World Bank. 2014 Land Cover Classification Of Accra, Ghana. 2014. https://datacatalog.worldbank.org/dataset/c-2014-land-cover-classification-accra-ghana
31 Volckens J, Quinn C, Leith D, *et al.* Development and evaluation of an ultrasonic personal aerosol sampler. *Indoor Air* 2017;**27**:409–16. doi:10.1111/ina.12318
32 Arku RE, Birch A, Shupler M, *et al.* Characterizing exposure to household air pollution within the Prospective Urban Rural Epidemiology (PURE) study. *Environ Int* 2018;**114**:307–17. doi:10.1016/j.envint.2018.02.033
33 Pillarisetti A, Carter E, Rajkumar S, *et al.* Measuring personal exposure to fine particulate matter (PM 2.5) among rural Honduran women: A field evaluation of the Ultrasonic Personal Aerosol Sampler (UPAS). *Environ Int* 2019;**123**:50–3. doi:10.1016/j.envint.2018.11.014
34 Bulot FMJ, Johnston SJ, Basford PJ, *et al.* Long-term field comparison of the performances of multiple low-cost particulate matter sensors in an urban area. *Sci Rep* 2019;:1–16. doi:10.5281/ZENODO.2531601
35 Malings C, Tanzer R, Hauryliuk A, *et al.* Fine particle mass monitoring with low-cost sensors: Corrections and long-term performance evaluation. *Aerosol Sci Technol* 2019;**0**:1–15. doi:10.1080/02786826.2019.1623863
36 Sather M, Slonecker T, Mathew J, *et al.* Evaluation of ogawa passive sampling devices as an alternative measurement method for the nitrogen dioxide annual standard in El Paso, Texas. *Environ Monit Assess* 2007;**124**:211–21.
37 Rindleisch TC. A comparative evaluation of two sound level meters. 2018. doi:10.13140/RG.2.2.26395.31520
38 Hill AP, Prince P, Piña Covarrubias E, *et al.* AudioMoth: Evaluation of a smart open acoustic device for monitoring biodiversity and the environment. *Methods Ecol Evol* 2018;**9**:1199–

For peer review only - http://bmjopen.bmj.com/site/about/guidelines.xhtml

211. doi:10.1111/2041-210X.12955

39 Fairbrass AJ, Firman M, Williams C, *et al.* CityNet—Deep learning tools for urban ecoacoustic assessment. Methods Ecol. Evol. 2018. doi:10.1111/2041-210X.13114

40 Xu M, Hong B, Jiang R, *et al.* Outdoor thermal comfort of shaded spaces in an urban park in the cold region of China. *Build Environ* 2019;**155**:408–20. doi:10.1016/j.buildenv.2019.03.049

41 Morguí J-A, Gacia E, Grossi C, *et al.* Atmospheric Carbon Dioxide variability at Aigüestortes, Central Pyrenees, Spain. *Reg Environ Chang* 2018;**19**:313–24. doi:10.1007/s10113-018-1443-2

42 Council of the European Union. Directive of The European Parliament and of The Council of 25 June 2002 relating to the assessment and management of environmental noise. 2002. https://publications.europa.eu/en/publication-detail/-/publication/27d1a64e-08f0-4665-a258-96f16c7af072/language-en

43 Jeronimo M, Stewart Q, Weakley AT, *et al.* Analysis of black carbon on filters by image-based reflectance. *Atmos Environ* 2020;**223**:117300. doi:10.1016/j.atmosenv.2020.117300

44 Ogawa. NO, NO2, NOx and SO2 sampling protocol using the Ogawa sampler. 2006. http://ogawausa.com/wp-content/uploads/2017/11/prono-noxno2so206_206_1117.pdf

45 Wunderli JM, Pieren R, Habermacher M, *et al.* Intermittency ratio: A metric reflecting short-term temporal variations of transportation noise exposure. *J Expo Sci Environ Epidemiol* 2016;**26**:575–85. doi:10.1038/jes.2015.56

46 Larkin A, Geddes JA, Martin R V., *et al.* Global land use regression model for nitrogen dioxide air pollution. *Environ Sci Technol* 2017;**51**:6957–64. doi:10.1021/acs.est.7b01148

47 Barrington-Leigh C, Millard-Ball A. The world's user-generated road map is more than 80% complete. *PLoS One* 2017;**12**:e0180698. doi:10.1371/journal.pone.0180698

48 GooglePlaces API. https://developers.google.com/places/web-service/intro (accessed 4 Mar 2020).

49 Hoek G, Beelen R, de Hoogh K, *et al.* A review of land-use regression models to assess spatial variation of outdoor air pollution. *Atmos Environ* 2008;**42**:7561–78. doi:10.1016/j.atmosenv.2008.05.057

50 Khan J, Ketzel M, Kakosimos K, *et al.* Road traffic air and noise pollution exposure assessment – A review of tools and techniques. *Sci Total Environ* 2018;**634**:661–76. doi:10.1016/j.scitotenv.2018.03.374

51 Lee M, Brauer M, Wong P, *et al.* Land use regression modelling of air pollution in high density high rise cities: A case study in Hong Kong. *Sci Total Environ* 2017;**592**. doi:10.1016/j.scitotenv.2017.03.094

52 He B, Heal MR, Reis S. Land-use regression modelling of intra-urban air pollution variation in China: Current status and future needs. *Atmosphere (Basel)* 2018;**9**:1–19. doi:10.3390/atmos9040134

53 Saraswat A, Apte JS, Kandlikar M, *et al.* Spatiotemporal land use regression models of fine, ultrafine, and black carbon particulate matter in New Delhi, India. *Environ Sci Technol* 2013;**47**. doi:10.1021/es401489h

54 Weichenthal S, Hatzopoulou M, Brauer M. A picture tells a thousand…exposures: Opportunities and challenges of deep learning image analyses in exposure science and environmental epidemiology. *Environ Int* Published Online First: 2018. doi:10.1016/j.envint.2018.11.042

55 Redmon J, Divvala S, Girshick R, *et al.* You Only Look Once: Unified, Real-Time Object Detection. *Proc 2016 IEEE Conf Comput Vis Pattern Recognit (CVPR 2016)* 2015;:779–88. doi:10.1109/CVPR.2016.91

56 Lin TY, Maire M, Belongie S, *et al.* Microsoft COCO: Common objects in context. *Lect Notes Comput Sci (including Subser Lect Notes Artif Intell Lect Notes Bioinformatics)* 2014;**8693 LNCS**:740–55. doi:10.1007/978-3-319-10602-1_48

57 Liu W, Anguelov D, Erhan D, *et al.* SSD: Single shot multibox detector. *Lect Notes Comput*

For peer review only

For peer review only - http://bmjopen.bmj.com/site/about/guidelines.xhtml

Sci (including Subser Lect Notes Artif Intell Lect Notes Bioinformatics) 2016;**9905 LNCS**:21–37. doi:10.1007/978-3-319-46448-0_2

58 Kuznetsova A, Rom H, Alldrin N, *et al.* The Open Images Dataset V4: Unified image classification, object detection, and visual relationship detection at scale. 2018;:1–20.http://arxiv.org/abs/1811.00982

59 Garcia AL, et al. A Cloud-Based Framework for Machine Learning Workloads and Applications." IEEE Access 8. 2020.

60 Gemmeke J, Ellis D, Freedman D, *et al.* Audio set: An ontology and human-labeled dataset for audio events. *IEEE ICASSP* 2017.

61 Changsong Y, BArsim KS, Kong Q, *et al.* Multi-level attention model for weakly supervised audio classification. *arXiv Prepr arXiv* Published Online First: 2018. doi:1803.02353

62 Skamarock WC, Klemp JB. A time-split nonhydrostatic atmospheric model for weather research and forecasting applications. *J Comput Phys* 2008;**227**:3465–85.

63 Byun DW, Schere KL. Review of the governing equations, computational algorithms, and other components of the models-3 Community Multiscale Air Quality (CMAQ) modeling system. *Appl Mech Rev* 2006;**59**:51–77. doi:Doi 10.1115/1.2128636

64 Heist D, Isakov V, Perry S, *et al.* Estimating near-road pollutant dispersion: A model inter-comparison. *Transp Res Part D Transp Environ* 2013;**25**:93–105. doi:10.1016/j.trd.2013.09.003

65 Beddows A V., Kitwiroon N, Williams ML, *et al.* Emulation and Sensitivity Analysis of the Community Multiscale Air Quality Model for a UK Ozone Pollution Episode. *Environ Sci Technol* 2017;**51**:6229–36. doi:10.1021/acs.est.6b05873

66 Bergin MS, Milford JB. Application of Bayesian Monte Carlo analysis to a Lagrangian photochemical air quality model. *Atmos Environ* 2000;**34**:781–92. doi:10.1016/S1352-2310(99)00346-5

Author contributions

All the authors contributed to this work and have taken part in the academic discussion for writing the study protocol, drafting the article and revising it. SNC, ASA, MB, ME, JB, MT, AH, JM, ST, JN, JV, SA, EA, BB, RA gave substantial contributions to conception and design and acquisition of data. SNC, ASA, MB, ME, MT, RN, EM, JB, JW, AB, FK, SB, RA gave substantial contributions to the analysis plan for data; SNC, ASA, MB, ME, JB, RA drafted and revised the manuscript; and all authors reviewed the final version.

Funding

This work is supported by the Pathways to Equitable Healthy Cities grant from the Wellcome Trust [209376/Z/17/Z]. SC is supported by a Canadian Institutes for Health Research (CIHR) PhD

For peer review only - http://bmjopen.bmj.com/site/about/guidelines.xhtml

scholarship as well as an Imperial College President's PhD scholarship. The content of this manuscript is solely the responsibility of the authors and does not necessarily represent the official views of the funders.

Ethics Approval

The study is primarily interested in pollution and features of the environment and was exempt from seeking ethics approval at Imperial College London and the University of Massachusetts-Amherst and was given ethical approval at the University of Ghana (ECH 149/ 18-19).

Competing interests

None

For peer review only

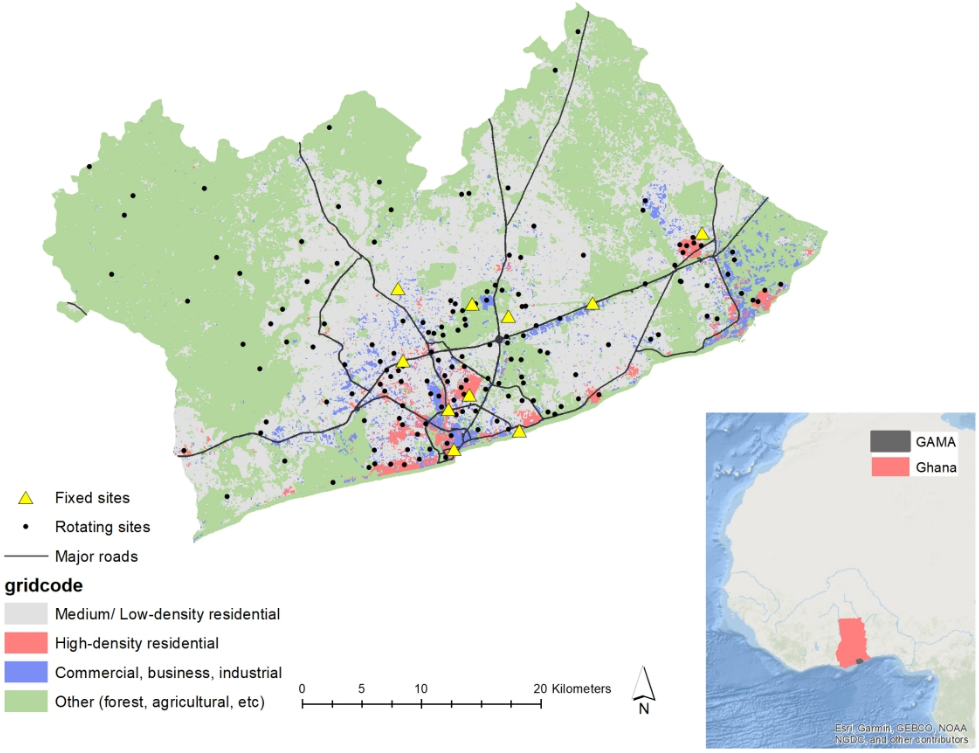

Figure 1. The Greater Accra Metropolitan Area (GAMA) and locations of the fixed and computer-generated (sampled) rotating sites. The road network data is from OpenStreetMap and the background land cover shapefile is from the World Bank (2014). The inset shows background maps of Africa and Ghana (ESRI), along with the GAMA boundary from Ghana Statistical Service. High-density residential indicates neighborhoods with small, crowded, irregular buildings and narrow unidentifiable unpaved roads such as in shantytowns and slums. Medium/ Low-density residential indicates neighborhoods with small regular planned buildings and indicate formal residential areas. Commercial/ business/ industrial indicates neighborhoods with large buildings that can be used for commercial, industrial, office, or warehouse purposes. Other indicates areas with large spaces of vegetation (e.g., dense forest), barren land (e.g., sand, soil), or water bodies.

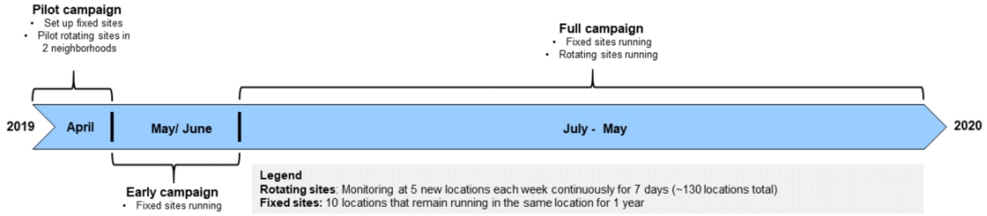

Figure 2. Timeline of measurement campaign. Weekly measurements consist of continuous (PM2.5 air concentration, noise levels, meteorological conditions, audio, and imagery) and integrated (PM2.5 and NOx concentration) samples. We chose weekly integrated samples for PM2.5 and NOx for logistical reasons (cost and time) as well as lessons from a previous study that showed relatively high temporal correlation between daily measurements [8].

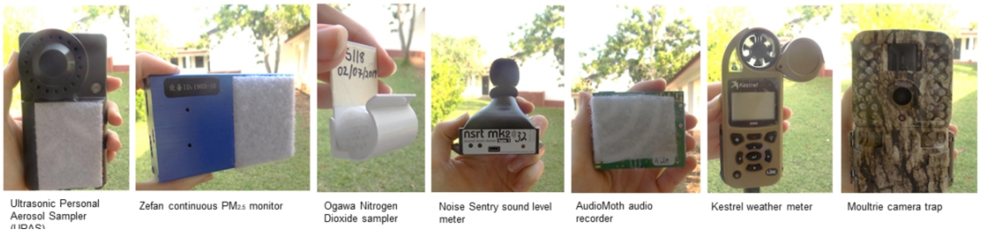

Figure 3. Images of environmental monitoring equipment.

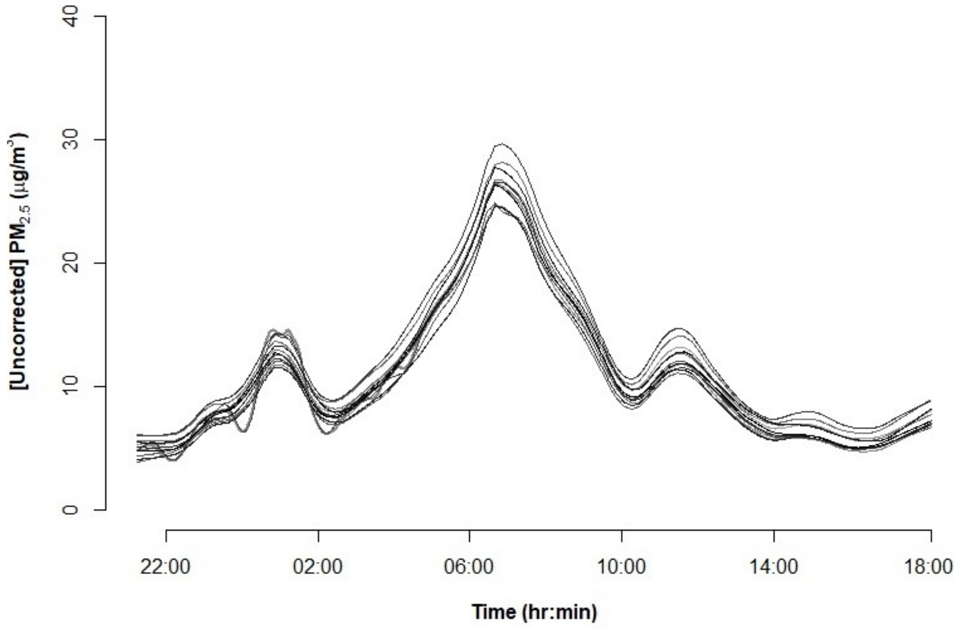

Figure 4. Smoothed time series of minute-by-minute PM2.5 from 15 co-located real-time Zefan monitors in Accra. The levels were neither corrected for relative humidity or against integrated filter-based data.

210x144mm (96 x 96 DPI)

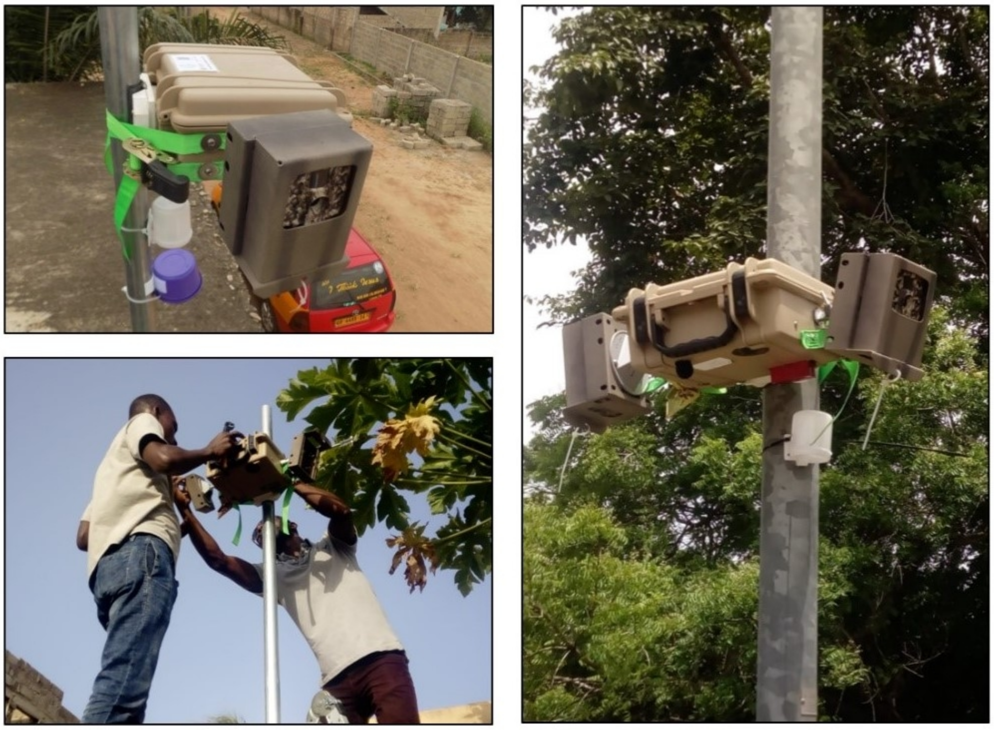

Figure 5. Deployment of the pollution measurement equipment.

265x195mm (96 x 96 DPI)

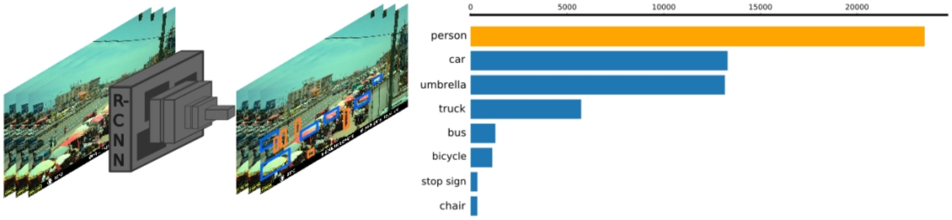

Figure 6. Illustration of how object detection models and street-level imagery can be combined from the Accra campaign data to identify potential correlates of air and noise pollution in the imagery. Information recorded on the bottom of the images includes the date and time, camera name, and the ambient temperature.

High-resolution spatiotemporal measurement of air and environmental noise pollution in sub-Saharan African cities: Pathways to Equitable Health Cities Study protocol for Accra, Ghana

Supplementary Information 1

Quality Assurance and Quality Control Protocol

The field team calibrate equipment prior to each use. Specifically, the UPAS mass flow sensor maintains a steady sampling flow rate over time by internally measuring changes in pressure drop across the filter media. But as part of our quality assurance process, the flow rates are manually checked with a TSI Mass Flowmeter (4000 Series) for possible flow drift prior to and immediately after each monitoring session. Monitors are adjusted as necessary prior to the next deployment. Following a previous protocol used in the same setting [1], samples will be considered valid only if the average flow rate is within 10% of the intended rate of 1 lpm, and the UPAS operated for ≥ 85% of the 7-day measurement period. Additionally, the SLMs are calibrated prior to each monitoring session with a CA114 sound calibrator at 94.0 dB ±0.3 dB and 1000Hz ±0.5% (Convergence Instruments, Canada). If an instrument is consistently reading a calibration offset ±1 dBA, the SLM is pulled out of commission and tested and the data from that session considered invalid.

In order to understand the extent of potential filter and diffusion pad contamination from handling procedures, we collect field blanks at 20% of our sites for filter based $PM_{2.5}$ and NO_x and NO_2 samples. Blank $PM_{2.5}$ samples are prepared as regular samples in the field lab, brought to the field sites, and deployed in the same way as the regular sample, but without the pump being turned on. NO_x/NO_2 blanks are brought to the field sites but not exposed to air in their sealed canisters. During analysis, information from the blank samples will be used to account for residual contamination from the laboratory work, transportation, and field handling processes, which in a previous study in Accra was minimal [1]. We will assess the mean absolute difference of the pre- and post-sampling weights of the blank samples; mean weights within 10 ug will be considered valid [1]. Also, final filters weights will be checked against the limit of detection, computed using the blanks, to be sure all valid samples are above this limit.

We will assess the accuracy and precision of our monitors by conducting **pre-campaign** side-by-side monitoring sessions between all our instruments of the same type (precision) and our instruments next to reference grade or higher-grade monitors (accuracy).

- Prior to field deployment, we tested minute-by-minute monitor-monitor precision for the continuous $PM_{2.5}$ monitors by running all of our monitors alongside each other over a 24-hour period at the University of Ghana, Legon campus, with average relative humidity (RH) (~ 78%) and temperature (29 °C) representative of the city. The continuous $PM_{2.5}$ measurements had good agreement and were within 2-3 ug/m^3 of each other. The continuous $PM_{2.5}$ ZeFan monitor uses the Plantower sensor (model PMS7003) which has been validated in previous studies against a TEOM 1400a analyser and tested for durations ranging from 6 months to a year in various environmental conditions [2,3].
- The filter-based UPAS monitor has been evaluated in previous laboratory and field settings against a federal reference monitor (URG-2000-30EGN-A; URG Corp., USA), personal environmental monitor (PEM 761 - 203; SKC, Inc., USA) and Harvard Impactors, respectively

and has proven valid for ambient, household, and personal monitoring in a typical tropical climate as our study [4–6].

- Our pre-campaign tests of SLM monitor-monitor precision showed good agreement. There was only a 0.5 dBA difference between the monitoring period median values ($LAeq_{1min}$) for 50% of monitors within the IQR bounds around the overall median (25%-75%) and a 1.7 dBA difference between the two monitors with the highest and lowest monitoring period median values. The monitor-monitor precision test was done in Accra and SLMs were exposed 16hrs to multiple sound environments similar to what we would expect during the full monitoring campaign. Our Type II Noise Sentry SLMs were also validated in a separate aircraft noise study conducted in San Francisco against a Type I industry standard instrument (DUO 01dB) [7], and the agreement was high (mean and median second by second difference between the instruments was -0.42 and -0.38 dBA, respectively).

In addition to the pre-campaign monitor-monitor precision tests and accuracy checks, we will collect duplicate samples at 20% of our sites and conduct **mid and post-campaign** precision tests to check their sensitivity over time and accuracy checks with reference grade monitors.

- To understand the extent to which each type of monitor provides consistent measurements among all the units used in the campaign, we are also collecting duplicate samples from co-located instruments at 20% of our rotating measurement sites. Duplicate samples will be evaluated from 20% of sites during the course of the campaign and faulty and malfunctioning instruments will be pulled from the field and data potentially removed from analysis if mean absolute difference between duplicate measurement is > 10 ug/m^3 [1] or >2 dBA ($LAeq_{24hr}$).
- We will additionally co-locate all of our monitors side-by-side for mid and post campaign precision tests for a 1-week period to assess instrument drift over time. Data will be considered invalid if the mean absolute difference between daily/ weekly $PM_{2.5}$ and $LAeq_{24hr}$ measurements differ by > 10 ug/m^3 [1] or >2 dBA.
- Since light-scattering techniques only infer PM mass from detecting particle number concentrations and are impacted by weather conditions (i.e. RH and temperature), their estimates of mass concentration are inexact. Thus, we will co-locate the ZeFan monitors with a U.S. federal equivalent continuous monitor Met One BAM 1020 at three sites, each with unique source influence in Accra for a week at the end of the campaign and adjust the minute-by-minute continuous PM records for impact of relative humidity and then their average against the co-located integrated $PM_{2.5}$ concentrations from UPAS.

The real-time data will be inspected weekly by the field team as it is downloaded from the instruments. Potential implausible values will be identified by inspecting all values that are 5-standard deviations above or below the site and day (or week for filter-based $PM_{2.5}$ and NO_x/NO_2) specific mean value. For the filter based $PM_{2.5}$ data, potentially implausible values will be checked against the monitor run time, weighed mass value, and flow rate. The log sheets will be checked to see if any information on instrument malfunction or other irregularities was noted for the continuous $PM_{2.5}$ and SLM monitors. Values deemed erroneous will be dropped from analysis. Additionally, since monitors are swapped every week, sometimes an entire week of data might be erroneous if the instrument is malfunctioning or if calibration did not occur correctly. We will identify outlier weeks by plotting timeseries of a month worth of data to identify any potential implausible weeks of data and conduct instrument checks, review log sheets, and drop or correct data as needed. Finally, all real-time instruments will have their first 5 minutes of data dropped to allow the instruments to stabilize and the data further trimmed to match the exact monitoring session start and end date and time as recorded by the field team on the data log forms.

For peer review only

References

1 Dionisio K, Arku RE, Hughes AF, *et al.* Air Pollution in Accra Neighborhoods: Spatial, Socioeconomic, and Temporal Patterns. *Environ Sci Technol* 2010;**44**:2270–6. doi:10.1021/es903276s

2 Bulot FMJ, Johnston SJ, Basford PJ, *et al.* Long-term field comparison of the performances of multiple low-cost particulate matter sensors in an urban area. *Sci Rep* 2019;:1–16. doi:10.5281/ZENODO.2531601

3 Malings C, Tanzer R, Hauryliuk A, *et al.* Fine particle mass monitoring with low-cost sensors: Corrections and long-term performance evaluation. *Aerosol Sci Technol* 2019;**0**:1–15. doi:10.1080/02786826.2019.1623863

4 Volckens J, Quinn C, Leith D, *et al.* Development and evaluation of an ultrasonic personal aerosol sampler. *Indoor Air* 2017;**27**:409–16. doi:10.1111/ina.12318

5 Arku RE, Birch A, Shupler M, *et al.* Characterizing exposure to household air pollution within the Prospective Urban Rural Epidemiology (PURE) study. *Environ Int* 2018;**114**:307–17. doi:10.1016/j.envint.2018.02.033

6 Pillarisetti A, Carter E, Rajkumar S, *et al.* Measuring personal exposure to fine particulate matter (PM 2.5) among rural Honduran women: A field evaluation of the Ultrasonic Personal Aerosol Sampler (UPAS). *Environ Int* 2019;**123**:50–3. doi:10.1016/j.envint.2018.11.014

7 Rindleisch TC. A comparative evaluation of two sound level meters. 2018. doi:10.13140/RG.2.2.26395.31520

For peer review only
